# Rise of cGMP by partial phosphodiesterase-3A degradation enhances cardioprotection during hypoxia

**DOI:** 10.1016/j.redox.2021.102179

**Published:** 2021-11-06

**Authors:** Nadja I. Bork, Anna Kuret, Melanie Cruz Santos, Cristina E. Molina, Beate Reiter, Hermann Reichenspurner, Andreas Friebe, Boris V. Skryabin, Timofey S. Rozhdestvensky, Michaela Kuhn, Robert Lukowski, Viacheslav O. Nikolaev

**Affiliations:** a Institute of Experimental Cardiovascular Research, University Medical Center Hamburg-Eppendorf, Hamburg, Germany; b DZHK (German Center for Cardiovascular Research), Partner Site Hamburg/Kiel/Lübeck, Hamburg, Germany; c Department of Pharmacology, Toxicology and Clinical Pharmacy, Institute of Pharmacy, University of Tübingen, Tübingen, Germany; d Department of Cardiovascular Surgery, University Heart & Vascular Center Hamburg, Hamburg, Germany; e Physiologisches Institut, University of Würzburg, Würzburg, Germany; f Core Facility Transgenic Animal and Genetic Engineering Models (TRAM), University of Münster, Münster, Germany

**Keywords:** Cardiomyocyte cGMP, FRET biosensor, Hypoxia, Ischemia/reperfusion, Phosphodiesterase

## Abstract

3′,5′-cyclic guanosine monophosphate (cGMP) is a druggable second messenger regulating cell growth and survival in a plethora of cells and disease states, many of which are associated with hypoxia. For example, in myocardial infarction and heart failure (HF), clinical use of cGMP-elevating drugs improves disease outcomes. Although they protect mice from ischemia/reperfusion (I/R) injury, the exact mechanism how cardiac cGMP signaling is regulated in response to hypoxia is still largely unknown. By monitoring real-time cGMP dynamics in murine and human cardiomyocytes using *in vitro* and *in vivo* models of hypoxia/reoxygenation (H/R) and I/R injury combined with biochemical methods, we show that hypoxia causes rapid but partial degradation of cGMP-hydrolyzing phosphodiesterase-3A (PDE3A) protein via the autophagosomal-lysosomal pathway. While increasing cGMP in hypoxia prevents cell death, partially reduced PDE3A does not change the pro-apoptotic second messenger 3′,5′-cyclic adenosine monophosphate (cAMP). However, it leads to significantly enhanced protective effects of clinically relevant activators of nitric oxide-sensitive guanylyl cyclase (NO-GC). Collectively, our mouse and human data unravel a new mechanism by which cardiac cGMP improves hypoxia-associated disease conditions.

## Graphical abstract

**Figure:**
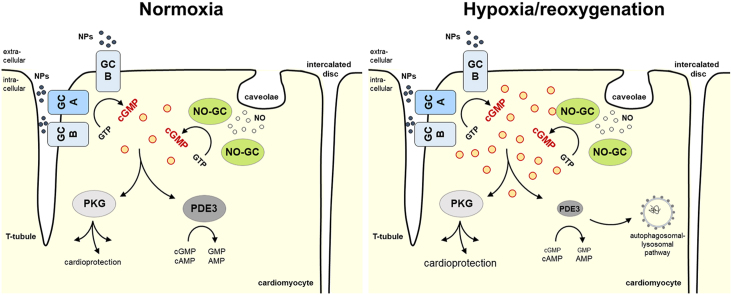

## Abbreviations

cAMP: 3′,5′-cyclic adenosine monophosphate
cGMP: 3′,5′-cyclic guanosine monophosphate
FRET: Förster resonance energy transfer
HF: heart failure
H/R: hypoxia/reoxygenation
I/R: ischemia/reperfusion
NO-GC: nitric oxide-sensitive guanylyl cyclase
PDE3A: phosphodiesterase 3A

## 1 Introduction

Hypoxia is a condition of oxygen imbalance, which is common for many diseases such as ischemic heart disease, heart failure (HF), and various cardiomyopathies [1,2]. It can cause serious pathological consequences such as impaired Ca^2+^ handling, and osmotic stress, which can result in contractile dysfunction, but also cardiac cell death [3]. 3′,5′-cyclic guanosine monophosphate (cGMP) is an important second messenger with a widely accepted cardioprotective function. In cardiomyocytes, cGMP is synthesized by either nitric oxide-sensitive guanylyl cyclases (NO-GCs) or particulate GCs, which are receptors for natriuretic peptides [4]. cGMP levels are controlled by the hydrolytic activity of multiple cGMP-degrading phosphodiesterases (PDEs), including PDE1, 2, 3, 5, and 9, whereby PDE3 as the major PDE family expressed in human heart is responsible for basal cGMP hydrolysis [5,6]. cGMP signaling is highly compartmentalized in so called cGMP microdomains orchestrated by local pools of GCs and PDEs [7,8]. Already small changes in cGMP-generating or degrading enzyme expression or activity can affect this balance and lead to pathological events [9,10].

The use of cGMP-elevating drugs has already shown promising results in various clinical trials. For example, the recently published results from the VICTORIA trial showed that the NO-GC stimulator vericiguat improves clinical outcome in patients suffering from worsening HF with reduced ejection fraction (HFrEF) [11] and led to its recent clinical approval. However, it is mechanistically not understood why specifically this group of patients with acutely worsening HF profit from vericiguat. Likewise, neprilysin inhibitors, which increase natriuretic peptide levels in the systemic circulation and cGMP in myocytes, used together with the angiotensin receptor blocker valsartan, are beneficial in patients with chronic HF as shown in PARADIGM-HF [12] and several other trials which led to their approval for the treatment of patients with HFrEF [13]. Recently, we have also demonstrated the cardioprotective effect of cGMP-elevating agents such as NO-GC stimulators, activators, and PDE inhibitors in an *in vivo* mouse model of ischemia/reperfusion (I/R) injury [[14], [15], [16]]. However, the mechanistic reason for beneficial effects of cGMP especially in ischemic heart disease is not well understood.

Despite these large body of pre-/clinical evidence, it remains largely unclear how the cGMP signaling cascade itself and its regulation are affected by hypoxia/reoxygenation (H/R) injury and what could be the functional outcomes of this interaction. One possible reason for this gap of knowledge could be the lack of appropriate cGMP imaging techniques, which can reliably monitor real-time cGMP dynamics under physiological and pathophysiological conditions in cells and animal models. In recent years, biosensors based on Förster resonance energy transfer (FRET) have become the state-of-the-art to study real-time cGMP dynamics in cardiac cells. With the development of the cGMP-FRET biosensor red-cGES-DE5 [5,17] it became possible to monitor and quantify cGMP dynamics in real-time in adult mouse and rat cardiomyocytes under various disease conditions [5,[17], [18], [19]].

To study the effects of H/R and I/R injury on the cGMP signaling cascade, we used this biosensor and biochemical techniques for cGMP measurements in isolated murine and human ventricular cardiomyocytes exposed to *in vitro* H/R, as well as a Langendorff model of global anoxic injury and a murine model of cardiac I/R injury *in vivo*. We could identify a novel role for PDE3A in the regulation of cardiomyocyte cGMP under these pathological conditions by showing that hypoxia and ischemia lead to acute downregulation of this PDE at the protein level through the action of autophagosomes, as a consequence of which basal and NO-GC stimulated cGMP levels can be increased to convey cardioprotection.

## 2 Material and methods

Detailed methods can be found in the Supplementary material.

### 2.1 Animal models

*FRET sensor mice.* Transgenic mice carrying the FRET-based cGMP biosensor red-cGES-DE5 under control of the α-myosin heavy chain (αMHC) promoter [5] and mice expressing the FRET-based 3′,5′-cyclic adenosine monophosphate (cAMP) sensor Epac1-camps [20] have been previously established on the FVB/N background. They were backcrossed at least 10 times to C57BL6/J background before starting the study.

*Cardiomyocyte-specific NO-GC knockout/red-cGES-DE5 mice.* Mice with cardiomyocyte-specific deletion of NO-GC (CM-NOGC-KO, on C57BL6/J background) [15] were crossbred to red-cGES-DE5 transgenic mice to generate CM-NOGC-KO/red-cGES-DE5 and respective control littermates (CM-NOGC-CTR/red-cGES-DE5) expressing the cGMP biosensor on the NO-GC knockout background upon Cre-mediated recombination (under control of the αMHC promoter) in cardiomyocytes.

*Generation of Pde3a conditional KO mouse model.* The *Pde3a* conditional KO (cKO) mouse model was generated using “one-step” strategy described previously [21]. In brief, mouse fertilized oocytes were microinjected with CRISPR-Cas9 complexes and long donor DNA template, containing two *LoxP* sites. Using bioinformatic analysis, we predicted that deletion of the Pde3a exon 12 would result in a translational frameshift and generation of premature stop codons and, subsequently, inactivation to the Pde3a through the Nonsense-mediated mRNA Decay (NMD) mechanism. We have designed a donor DNA fragment harboring exon - intronic regions of *Pde3a* flanked by *LoxP* sites with short 60 nt homology arms (Supplementary Figs. S9A and C). Efficient CRISPR-Cas9 complexes were selected after microinjection into the cytoplasm of fertilized mouse oocytes *in vivo*, as previously described [21]. Positively targeted F0 mouse founders, born after microinjection procedure were selected and crossed with wildtype C57BL6 animals to produce independent heterozygous mouse lines. Extensive PCR, sequencing and Southern blot analyses identified germ-line transmitted F1 mice with correctly targeted *Pde3a* exon 12 genomic locus (Supplementary Figs. S9D,E,F), which were used to establish independently derived mouse lines that later were backcrossed to C57BL6/J background and bred with αMHC-Cre deleter mice on the same background for constitutive tissue-specific *Pde3a* deletion in CMs.

### 2.2 Mouse cardiomyocyte isolation

Adult ventricular mouse cardiomyocytes were freshly isolated as described previously [22]. In short, hearts were perfused retrogradely via the aorta with an enzyme solution containing liberase DH (0.04 mg/mL) and trypsin (0.008%). Afterwards, cells were recalcified, plated on laminin-coated dishes, and cultured in murine myocyte culture medium (minimum essential medium containing bovine serum albumin (BSA) (0.1%), 2,3-butanedione monoxime (BDM) (10 mM), l-glutamine (1%), insulin-transferrin selenium supplement (1x), and penicillin/streptomycin (1%). For *in vitro* hypoxia with glucose deprivation experiments, medium containing Dulbecco's Modified Eagle Medium without glucose containing BSA (0.1%), BDM (10 mM), l-glutamine (1%), penicillin/streptomycin (1%), and l-glucose (5.55 mM) was used instead of murine CM culture medium.

*Human tissue samples and cardiomyocyte isolation.* Human ventricular tissue samples were obtained from patients with/without HF at the time of surgery. Clinical characteristics of patients are described in the Supplemental Methods section. Fresh tissue samples were used for cardiomyocyte isolation as previously described [23]. After isolation, cells were resuspended in human myocyte culture medium (minimum essential medium containing creatine (12 mM), taurine (20 mM), penicillin/streptomycin (1%), l-glutamine (1%), BSA (0.1%), blebbistatin (24 μM), and fetal calf serum (FCS) (2.5%)), and plated on laminin-coated dishes and 2h later, FCS-free culture medium was added.

### 2.3 Models of hypoxia/reoxygenation (H/R) injury

*In vitro H/R injury model.* A modular incubator chamber (MIC-101, Billups-Rothenberg, Inc.) flushed with N_2_ and CO_2_ was used to induce H/R injury in freshly isolated murine or human ventricular myocytes. Final O_2_ concentration in the chamber was 1% (94% N_2_, 5% CO_2_, 1% O_2_). Hypoxia duration was 90 min or 4h. Reoxygenation was performed for 2 h under standard conditions (5% CO_2_, 37 °C) (Supplementary Fig. S1A).

*Langendorff model of global anoxic injury.* Mouse hearts were perfused retrogradely and under constant pressure (80 mmHg) in a Langendorff system (Harvard apparatus, Hugo Sachs Elektronik) with pre-warmed (37 °C) and carbogen aerated (95% O_2_, 5% CO_2_) modified Krebs-Henseleit solution (NaCl, 118 mM; KCl, 4.7 mM; MgSO_4_, 0.8 mM; NaHCO_3_, 25 mM; KH_2_PO_4_, 1.2 mM; glucose 5 mM; Na-pyruvate, 24 mM; CaCl_2_,2.5 mM; pH 7.4). Right ventricular pacing at 530 beats per minute was done with an octapolar electrophysiology catheter. Cardiovascular parameters were monitored with a digital data acquisition system (STG 4002, Multi channel systems). Hearts were perfused with non-oxygenated modified Krebs-Henseleit solution (37 °C) for 30 min to induce global anoxia (Supplementary Fig. S1B).

*Open chest model of ischemia/reperfusion (I/R) injury.* For studying I/R injury, an acute open-chest in situ model of ischemia and reperfusion injury was used as described previously [14,15,24]. Left coronary artery was occluded for 30 min, reperfusion was allowed for 10 min (Supplementary Fig. S1C). Tissue from the area-at-risk (AAR) defined as percentage of total heart area was determined by a double staining technique with Evans Blue and triphenyltetrazolium chloride (TTC) as previously described [14,15] (Supplementary Fig. S6A). For further experiments (Supplementary Fig. S6B) the conditioned hearts were divided into two regions “I/R-affected” and “I/R unaffected” without prior staining. I/R affected myocardium mainly consists of cardiac tissue from the AAR and just a minor number of cells, not affected from the ligation (mainly derived from the right ventricle). The stained heart in Supplementary Fig. S6B illustrates the distinction I/R-affected vs. I/R unaffected and AAR vs. area-not-at-risk (ANAR).

### 2.4 Statistics

Statistical analysis was performed using GraphPad Prism 6 and R 3.3.2 software. Data are presented as mean ± SEM. Normal distribution was tested with the D'Agostino & Pearson omnibus normality test. For pairwise comparison, unpaired or paired *t*-test in case of normal distribution and Mann-Whitney or Wilcoxon matched-pairs signed rank test in case of skewed distribution were used, respectively. 2-way ANOVA with Turkey's multiple comparisons test, Dunn's multiple comparisons test or Bonferroni's multiple comparisons test were used for experiments with 2-factor design. Measurements done with several cells from different mice/patients were assessed with mixed ANOVA followed by χ^2^ test. Values of p < 0.05 for all tests were considered statistically significant.

### 2.5 Study approval

All animal work was done with both male and female mice according to national and international animal welfare guidelines and approved by the local animal welfare authorities BGV Hamburg (ORG 741, ORG 1010 and N106/2020), the Ethics Committee for Animal Research (Regierungspraesidium Tuebingen), and the LANUV Nordrhein-Westfalen.

For all human ventricular tissue samples, patients provided written informed consent. The study followed the Declaration of Helsinki and was approved by the Ethical Committees of the Aerztekammer Hamburg (WF-088/18).

## 3 Results

### 3.1 *In vitro* H/R injury increases basal cGMP levels in murine ventricular myocytes

**Fig. 1:**
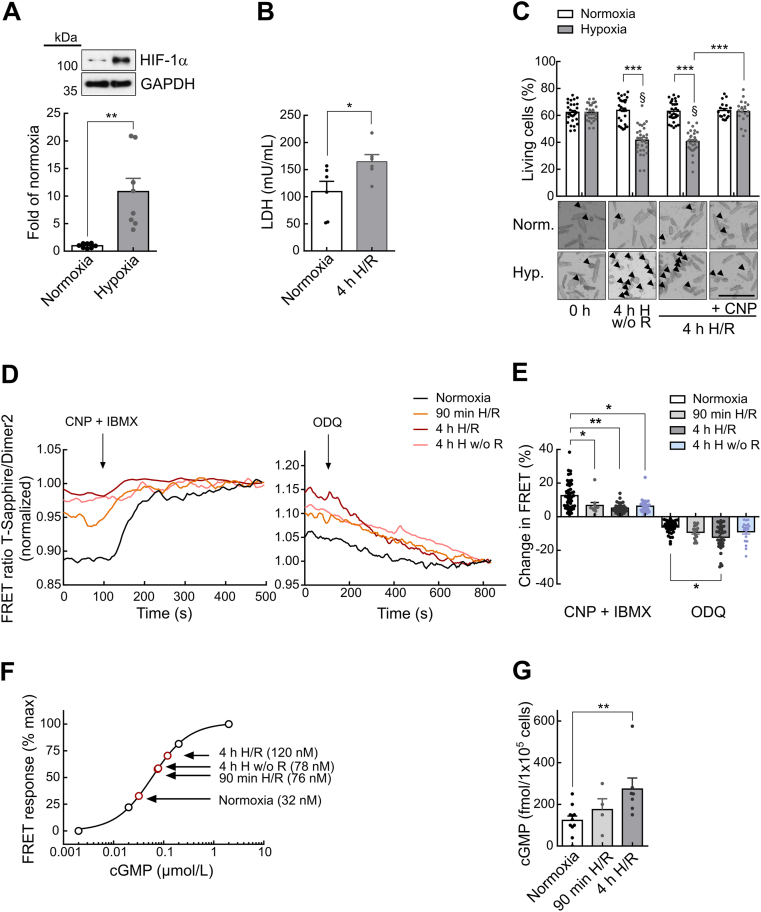
***In vitro* hypoxia/reoxygenation injury increases basal cGMP levels in murine ventricular myocytes. A** Representative immunoblots and quantification for hypoxia inducible factor 1α (HIF-1α) in cardiomyocytes after hypoxia/normoxia treatment with GAPDH as loading control (n = 8 mice). **B** Lactate dehydrogenase (LDH) release in cardiomyocytes after hypoxia/normoxia treatment (n = 6 mice). **C** Light microscopy images and quantification of cardiomyocytes taken before induction of hypoxia (0h), 4h after hypoxia without reoxygenation (4h H w/o R) and after 2h of subsequent reoxygenation (4h H/R). Some cells were treated with CNP (100 nM) during 4h H/R. Black arrowheads indicate dead cardiomyocytes. Scale bar, 250 μm (n = 3–5 mice, 6 pictures each). **D** red-cGES-DE5 cardiomyocytes were used for cGMP-FRET measurements after 90 min or 4h hypoxia treatment with/without 2h of reoxygenation (90 min H/R, 4h H/R, 4h H w/o R), compared to normoxic control. Maximal cGMP responses were reached by using saturating concentrations of CNP (1 μM) together with the pan-PDE inhibitor IBMX (100 μM), minimal cGMP levels by using the NO-GC inhibitor ODQ (50 μM). Representative FRET traces are shown. **E** Quantification of the FRET responses shown in D. Number of measured cardiomyocytes/mice were: normoxia_CNP + IBMX_ = 49/9; 90 min H/R_CNP + IBMX_ = 10/3; 4h H/R_CNP + IBMX_ = 35/6; 4h Hw/oR_CNP + IBMX_ = 25/3; normoxia_ODQ_ = 39/9; 90 min H/R_ODQ_ = 15/3; 4h H/R_ODQ_ = 34/6; 4h Hw/oR_ODQ_ = 19/3. **F** Averaged intracellular cGMP concentrations calculated from data in D using a biosensor *in vitro* calibration curve [18]. **G** cGMP measurements by immunoassay in cardiomyocytes exposed to normoxia, 90 min H/R or 4h H/R (n = 4–11 mice). Data in **A,B** and **G** were analyzed with Mann-Whitney test, in **C** by 2-way ANOVA with Turkey's multiple comparisons test, data in **E** with mixed ANOVA followed by *χ*^2^ test. *p < 0.05, **p < 0.01, ***p < 0.001 for normoxia vs H/R. §p < 0.001 vs respective t = 0h group. (For interpretation of the references to colour in this figure legend, the reader is referred to the Web version of this article.)

We first studied the susceptibility of isolated adult murine cardiomyocytes to H/R injury in an *in vitro* H/R model (Supplementary Fig. S1A). Protein levels of the hypoxia inducible factor 1α (HIF-1α) revealed a rapid and robust upregulation after 4 h of hypoxia (10.8 ± 2.4-fold; n = 8, compared to normoxic control) (Fig. 1A). We did this treatment without reoxygenation because under normal oxygenated conditions the half-life of HIF-1α protein is within several minutes [25]. Lactate dehydrogenase (LDH) release was significantly increased after 4h H/R treatment compared to normoxia, indicating cell damage caused by hypoxia (Fig. 1B). Quantification of living and dead cardiomyocytes during H/R showed a significantly reduced survival during 4h hypoxia without reoxygenation (41.7 ± 1.9% living cells vs. 63.7 ± 1.7% at normoxic control conditions) as well as after 2h of subsequent reoxygenation (40.5 ± 1.5% living cells vs. 63.0 ± 1.3% in normoxic control) (Fig. 1C). C-type natriuretic peptide (CNP), acting through activation of membrane GC, applied during H/R could significantly reduce cell death (62.8 ± 2.1% living cells) as compared to the H/R injury group without CNP (Fig. 1C).

To analyze the effect of H/R injury on cardiomyocyte cGMP levels, we applied cGMP live cell imaging as well as cGMP immunoassay. To investigate whether the duration of hypoxia had an effect on the cGMP levels, we used two different time points, 90 min and 4 h of hypoxia, both followed by 2 h of reoxygenation. For live cell FRET responses, we used our previously established imaging protocol to measure basal cytosolic cGMP concentrations [22]. Cells were first treated with saturating concentrations of CNP plus the pan-PDE inhibitor 3-isobutyl-1-methylxanthine (IBMX) to record maximal FRET responses. Alternatively, cells were treated with the NO-GC inhibitor 1H-[1,2,4]oxadiazolo[4,3-a]quinoxalin-1-one (ODQ) to decrease cGMP levels to a minimum value measurable by the biosensor [5] (Fig. 1D and E). H/R treatment led to a decrease of the CNP/IBMX response as compared to normoxic control already after 90 min of hypoxia (normoxia: 12.6 ± 1.2%; 90 min H/R: 6.7 ± 1.8%; 4h H/R: 5.1 ± 0.5%), whereas the effect of ODQ was concomitantly increased (normoxia: 6.1 ± 0.6%; 90 min H/R: 9.2 ± 2.0%; 4h H/R: 12.1 ± 1.3%) (Fig. 1D and E). Using the concentration-response dependency of the red-cGES-DE5 biosensor protein to cGMP measured *in vitro* [18] we calculated basal cGMP levels of ∼32 nM in normoxia-treated cells (Fig. 1F). With hypoxia, cGMP levels increased by ∼2.4-fold at 90 min H/R and up to ∼4-fold after 4h H/R without reaching statistical significance between these two time points (Fig. 1F). Cells exposed to 4h of hypoxia without reoxygenation showed higher cGMP levels as compared to normoxia (∼78 nM vs. ∼32 nM) but not significantly different from cGMP levels induced by 90 min H/R (∼76 nM), suggesting that the rise of cardiomyocyte cGMP occurs already at hypoxia but continues to increase during the reoxygenation period (Fig. 1D,E,F).

Analysis of cGMP levels by conventional cGMP immunoassay confirmed significantly increased basal cardiomyocyte cGMP levels after 4h H/R (273.4 ± 53.0 fmol/10^5^ cells, n = 7) as compared to normoxic control (123.3 ± 20.9 fmol/10^5^ cells, n = 11). After 90 min H/R treatment, samples showed already a strong tendency toward upregulated cGMP levels (175.2 ± 52.1 fmol/10^5^ cells, n = 4) (Fig. 1G), which is compatible with live cell imaging results. Assuming a mean cell volume of approximately 20 000 fL [26], these values corresponds to ∼62 nM cGMP in normoxia, ∼88 nM after 90 min H/R and ∼137 nM cGMP after 4h H/R.

### 3.2 PDE3A levels are reduced in mouse cardiomyocytes after *in vitro* H/R injury due to protein instability

**Fig. 2:**
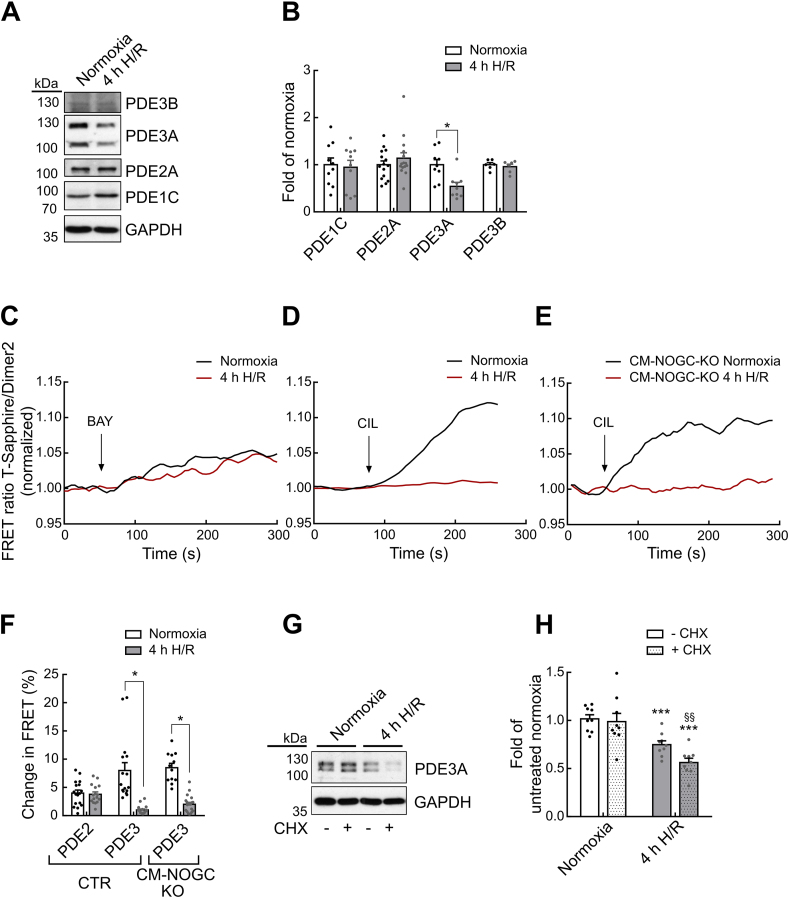
**cGMP degrading PDE3A protein is reduced after *in vitro* hypoxia/reoxygenation injury due to protein instability. A**, **B** Representative immunoblots (**A**) and quantification (**B**) for PDE1C, PDE2A, PDE3A (both long PDE3A1 and shorter PDE3A2 isoforms are detectable), and PDE3B protein in cardiomyocytes after 4h H/R. Protein levels were normalized to GAPDH (n = 6–15 mice per condition). **C**,**D** cGMP-FRET response to specific PDE inhibitors was measured in red-cGES-DE5 cardiomyocytes after 4h H/R or normoxia treatment. Representative traces showing effects of PDE2 inhibition with BAY 60–7550 (BAY, 100 nM) (**C**) and PDE3 inhibition with Cilostamide (CIL, 10 μM) (**D**). **E** FRET response of cardiomyocyte-specific NO-GC knockout (CM-NOGC-KO)/red-cGES-DE5 myocytes to the PDE3-selective inhibitor cilostamide (CIL, 10 μM) after exposure to 4h H/R or normoxia. **F** Quantification of cGMP-FRET response to specific PDE inhibition shown in C-E. Number of measured cardiomyocytes/mice were: normoxia_BAY_ = 17/3, 4h H/R_BAY_ = 16/3; normoxia_CIL_ = 16/4, 4h H/R_CIL_ = 18/4; CM-NOGC-KO normoxia_CIL_ = 12/3, CM-NOGC-KO 4h H/R_CIL_ = 20/3. **G** Myocytes were treated with cycloheximide (CHX, 50 μM) to inhibit protein synthesis and afterwards exposed to 4h H/R or normoxia. Vehicle treated myocytes were used as respective controls. Representative immunoblots for PDE3A protein levels. **H** Quantification of immunoblot experiments shown in G as fold of the untreated normoxia control group. n = 9 mice per condition. Data in **B** were assessed with Mann-Whitney test, in **F** with mixed ANOVA with *χ*^2^ test, in **H** with unpaired *t*-test. *p < 0.05 for 4h H/R vs normoxia in B and F. In H: ***p < 0.001 vs respective normoxia group and §§p < 0.01 vs untreated 4h H/R group. (For interpretation of the references to colour in this figure legend, the reader is referred to the Web version of this article.)

To uncover the mechanisms that lead to an increase in basal cGMP levels during H/R injury, we investigated the effect of H/R on cGMP degradation by PDEs and cGMP generation by GCs. The PDE3A protein abundance was significantly lower after 4h H/R (0.54 ± 0.09-fold expression as compared to normoxia, n = 9), whereas PDE1C, PDE2A, and PDE3B protein levels remained unchanged (Fig. 2A and B). Cyclic GMP generating GC-B and NO-GCα_1_ protein levels remained unchanged during 4h H/R, whereas, NO-GCβ_1_ was less abundant (0.54 ± 0.08-fold expression as compared to normoxia, n = 10) (Supplementary Figs. S2A and B). Transcript analysis of *Gucy1a1*, *Gucy1b1*, *Nrp1*, and *Npr2* expression after 4h H/R revealed no significant changes compared to normoxia. Accordingly, Pde3a mRNA is not significantly altered among the different treatment groups (Supplementary Fig. S2C), which strongly implies that the observed regulation of PDE3A by H/R occurs at the protein and/or activity level.

Since PDE3 has been shown to tightly regulate basal cGMP levels in murine cardiomyocytes [5], we analyzed its function by applying selective PDE inhibitors to H/R exposed cardiomyocytes. Interestingly, whereas PDE2 inhibition with the PDE2-selective inhibitor BAY 60–7550 (BAY) only slightly raised cGMP in normoxia (4.0 ± 0.5%), as well as in 4h H/R (3.8 ± 0.4%) treated cardiomyocytes (Fig. 2C,F), the selective PDE3 inhibitor cilostamide (CIL) strongly increased cGMP levels at normoxia (8.0 ± 1.4%), while this effect was abolished in 4h H/R exposed cardiomyocytes (1.0 ± 0.2%) (Fig. 2D,F).

Basal cGMP generation usually stems from NO-GC function [5], hence, we further investigated the NO-GC effect in H/R injury using Cre/loxP-based cardiomyocyte-specific NO-GC knockout mice [15] that expressed the red-cGES-DE5 cGMP biosensor in the same cell type i.e., in cardiomyocyte. Diminished FRET response to the NO-GC inhibitor ODQ in NO-GC knockout (KO) cardiomyocytes (CM-NOGC-KO -0.9 ± 0.1%) compared to NO-GC control cardiomyocytes (CM-NOGC-CTR: -6.1 ± 0.6%) demonstrated that the Cre-mediated deletion of NO-GC was of functional consequence for basal cGMP production (Supplementary Figs. S2D and E). cGMP levels were assessed by FRET imaging as described above (Supplementary Fig. S2F). cGMP concentrations in normoxic CM-NOGC-KO were ∼4 nM, but these values were still significantly increased with 4h H/R to ∼12 nM. Consistent with NO-GC proficient cardiomyocytes (Fig. 2D), the cGMP-FRET response to CIL was ablated in CM-NOGC-KO cardiomyocytes exposed to 4h H/R (2.0 ± 0.3%) as compared to normoxia treatment (8.5 ± 0.8%) (Fig. 2E and F). These findings suggest that the H/R-induced changes in the PDE3-sensitive cGMP pool were not primarily affected by the cellular NO-GC status.

Since PDE3A protein but not mRNA levels were reduced after H/R, we next hypothesized that protein stability of this PDE could be compromised under hypoxic conditions. To assess PDE3A protein stability, we performed cycloheximide (CHX) protein chase assay. Inhibition of translation did not alter PDE3A protein levels during normoxia (0.99 ± 0.09-fold compared to untreated normoxia, n = 9), whereas in CHX treated cardiomyocytes exposed to 4h H/R (0.56 ± 0.04-fold compared to untreated normoxia, n = 9) PDE3A protein levels significantly declined as compared to the respective cardiomyocyte samples that were treated for 4h H/R without CHX (0.75 ± 0.04-fold compared to untreated normoxia, n = 9) (Fig. 2G and H). This indicates rapid destabilization of the PDE3A protein during H/R injury.

Since the use of an *in vitro* 1% hypoxia model is limited in mimicking the near anoxic conditions in the heart after an ischemic injury, we assessed cell survival *in vitro* using a harsher model of hypoxic injury i.e., 1% of oxygen applied together with glucose deprivation, using cell culture medium in which physiological d-Glucose has been exchanged with non-metabolizable l-Glucose to maintain proper osmotic properties (Supplementary Fig. S3). Quantification of living and dead cardiomyocytes during hypoxic injury with concomitant glucose deprivation showed a significantly reduced survival after 4h of H w/o R as compared to cells cultured in presence of glucose (24.3 ± 1.4% vs. 43.4 ± 1.2% living cells). Similar effect was observed in cells after 4H/R treatment (28.7 ± 1.7% living cells with vs. 44.4 ± 2.2% living cells without glucose deprivation), indicating that hypoxia in combination with glucose deprivation has an even more severe effect on the survival of murine cardiomyocytes than hypoxia alone. CNP treatment could significantly reduce cell death in both groups (4h H/R + CNP: 61.2 ± 2.1%; 4h H/R/L-glucose + CNP: 58.2 ± 1.3% living cells). Interestingly, inhibition of PDE3 with its selective inhibitor cilostamide (CIL) increased cell death under normoxic conditions as previously reported [27]. However, cilostamide treatment significantly increased cell death in the hypoxia treated (4h H/R + CIL: 36.1 ± 3.3% living cells) but not in 4h H/R/l-glucose treated cardiomyocytes (30.7 ± 1.5% living cells) (Supplementary Fig. S3A). cGMP levels, assessed by FRET imaging as described above, showed significantly higher cGMP levels after 4h H/R/l-glucose (157.2 ± 13.1 nM vs. 98.1 ± 5.4 nM in 4h H/R and 20.5 ± 3.3 nM in normoxia), although FRET values were not significantly different between 4h H/R and 4h H/R/l-glucose groups (Supplementary Figs. S3B and C). Furthermore, we assessed PDE3A protein levels after 4h H/R with concomitant glucose deprivation. PDE3A protein was significantly less after 4h H/R/l-glucose treatment (0.08 ± 0.03-fold expression as compared to normoxia) as compared to 4h H/R only treatment (0.71 ± 0.08-fold expression as compared to normoxia) (Supplementary Fig. S3D).

### 3.3 *In vitro* H/R injury affects PDE3-mediated cGMP/cAMP crosstalk after β-adrenergic and natriuretic peptide co-stimulation

**Fig. 3:**
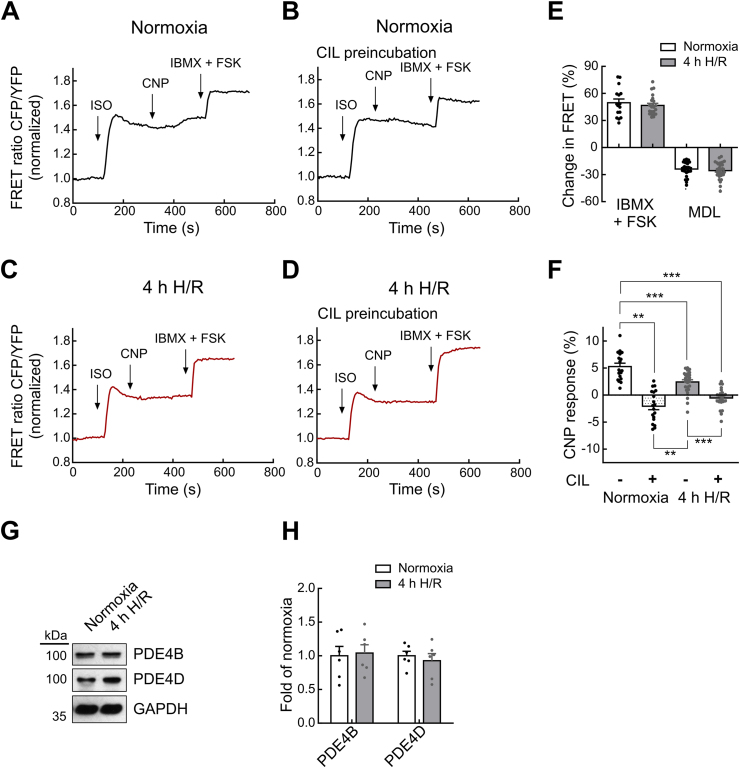
***In vitro* H/R injury affects PDE3-mediated cGMP/cAMP crosstalk after β-adrenergic and natriuretic peptide co-stimulation. A**-**D** Representative FRET recordings in myocytes expressing the cytosolic cAMP biosensor Epac1-camps. After prestimulation with the β-adrenergic receptor agonist isoprenaline (ISO, 100 nM), the natriuretic peptide CNP (100 nM) was added to measure cGMP/cAMP crosstalk. Maximal FRET response was evoked by the direct adenylyl cyclase activator forskolin (FSK, 10 μM) plus IBMX (100 μM) for unspecific PDE inhibition. Before FRET measurements, myocytes were exposed to either normoxia (**A**,**B**) or 4h H/R (**C**,**D**). Pretreatment with Cilostamide (CIL, 10 μM) for selective PDE3 inhibition (**B**,**D**) reduced CNP responses. **E** Minimal cAMP responses were reached by using the adenylyl cyclase inhibitor MDL-12,330A (MDL, 100 μM). Quantification of minimal/maximal signals to MDL or IBMX + FSK, respectively, under normoxia and 4h H/R. Number of measured cardiomyocytes/mice were: normoxia_IBMX + FSK_ = 16/4, 4h H/R_IBMX + FSK_ = 22/4; normoxia_MDL_ = 25/4, 4h H/R_MDL_ = 29/4. **F** Quantification of FRET responses to CNP (100 nM) shown in A-D. Number of measured cardiomyocytes/mice were: normoxia_CNP_ = 18/4, normoxia + CIL_CNP_ = 19/4, 4h H/R_CNP_ = 25/4, 4h H/R + CIL_CNP_ = 23/4. **G**, **H** Representative immunoblots (**G**) and quantification (**H**) of PDE4B and PDE4D proteins in cardiomyocytes after 4h H/R. Protein levels were normalized to GAPDH (n = 6 mice). Data in **E** and **F** were assessed with mixed ANOVA followed by *χ*^2^ test, in **H** with Mann-Whitney test. **p < 0.01, ***p < 0.001.

Since PDE3A has a dual-specific enzyme hydrolyzing both cGMP and cAMP and mediating their crosstalk after β-adrenergic stimulation [5], we next studied whether crosstalk between these second messengers was also affected by H/R. Therefore, isolated cardiomyocytes expressing cAMP-FRET biosensor Epac1-camps [20] were exposed to our *in vitro* H/R injury model. Single cell cAMP-FRET showed that isoprenaline (ISO), a β-adrenergic receptor agonist, raised cytosolic cAMP. In normoxia-treated cells, subsequent addition of CNP further increased cAMP levels (normoxia_CNP_: 5.3 ± 0.6% change in FRET) (Fig. 3A,F). As expected for the PDE3-dependent cGMP/cAMP crosstalk [5], this effect could be blocked by preincubation with CIL (normoxia + CIL_CNP_: 2.0 ± 0.7%) (Fig. 3B,F). We also stimulated the cells with the adenylyl cyclase activator forskolin (FSK) and the pan-PDE inhibitor IBMX to induce maximal cAMP-FRET response. Alternately, we used MDL-12,330A (MDL) to block cAMP production by inhibition of adenylyl cyclase (Fig. 3A,C,E). Cardiomyocytes exposed to 4h H/R treatment showed significantly reduced or completely blunted increase in cAMP after CNP (4h H/R_CNP_: 2.4 ± 0.4%; vs normoxia_CNP_: 5.3 ± 0.6%) (Fig. 3A,C,F). These data indicate that the functional impairment of PDE3A caused by H/R injury affects the CNP-induced raise in cAMP and thus the cGMP/cAMP crosstalk. We also tested whether 4h H/R had any effect on basal cAMP levels. Using the FSK/IBMX and MDL data (Fig. 3E) we calculated cAMP levels from FRET data as previously established [22]. Importantly, and in contrast to cGMP (Fig. 1F and G), under these experimental conditions, hypoxia did not lead to any significant change in basal cAMP concentrations (∼930 nM at normoxia and ∼1100 nM after 4h H/R). To ensure that H/R treatment has no effect on PDE4, which, besides PDE3, is mainly responsible for cAMP degradation [28], we determined the PDE4B and PDE4D protein levels. 4h H/R did not alter PDE4B and PDE4D abundance compared to normoxia (Fig. 3G and H).

### 3.4 Basal cGMP levels are increased during global anoxia and after acute open chest I/R injury due to PDE3A protein instability

The data shown so far have been collected in an *in vitro* model of H/R injury. To investigate the reproducibility of these results in a more physiologically relevant context, we first assessed a Langendorff model of global anoxic injury (Supplementary Fig. S1B). Hearts from red-cGES-DE5 transgenic mice were perfused with non-oxygenated buffer solutions to monitor cardiomyocyte-specific changes in cGMP-FRET during anoxic injury (Supplementary Figs. S4A and B). Since it is widely accepted that the pH of the myocardial tissue drops with ischemia [29] and some FRET sensors can be affected by changes in pH [30,31], we first made sure that the two fluorescent proteins T-Sapphire and Dimer2 in the red-cGES-DE5 sensor are not impaired by acidosis (Supplementary Fig. S5). While whole heart measurements under normoxic control conditions showed no change in cGMP-FRET ratio (Supplementary Fig. S4C), induction of anoxia caused a raise in cGMP-FRET indicating increased cGMP levels (Supplementary Fig. S4D). Even in hearts obtained from cardiomyocyte-specific NO-GC KO mice, cGMP levels increased during anoxia (Supplementary Fig. S4E). Data obtained from the isolated perfused heart assay imply that 30 min of anoxia and thus whole heart ischemia is per se an effective driver of basal cGMP, which increases under these conditions presumably through a PDE3A dependent mechanism.

**Fig. 4:**
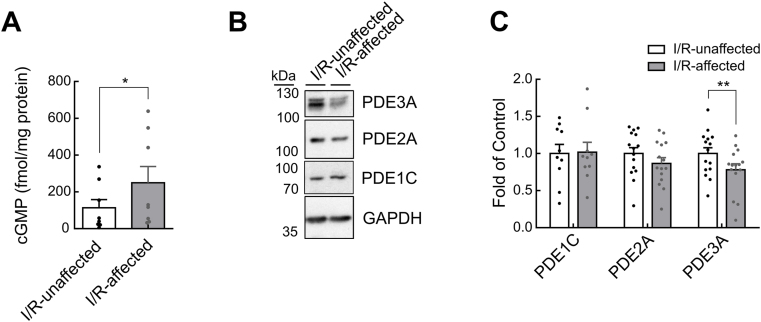
**Basal cGMP levels are increased in an acute open chest model of ischemia/reperfusion injury.** Ischemia/reperfusion (I/R) injury in mouse hearts was induced *in vivo* by left coronary artery occlusion for 30 min and 10 min of reperfusion. I/R affected tissue containing predominantly cells from the area-at-risk (AAR) and I/R-unaffected tissue from the area-not-at-risk (ANAR) of the same hearts were analyzed. See Supplementary Fig. 6 for details on tissue preparation. **A** cGMP immunoassay shows increased basal cGMP levels in I/R-affected region (n = 8). **B**, **C** Representative immunoblots (**B**) and analysis (**C**) of PDE1C, PDE2A, and PDE3A protein levels normalized to GAPDH (n = 10–15). Data were assessed with paired *t*-test. *p < 0.05, **p < 0.01 for I/R-unaffected vs I/R-affected.

Next, we used an acute open chest model of I/R injury (Supplementary Fig. S1C) to investigate changes in cGMP dynamics in living mice i.e. in an even more pathophysiologically relevant context. The area-at-risk (AAR) defined as percentage of total heart area was determined as a marker for the reproducibility of the surgery (Supplementary Fig. S6). cGMP immunoassay in mouse heart tissues collected after the I/R protocol showed significantly increased cGMP levels in the AAR containing the infarct zone (I/R-affected: 249.7 ± 88.6 fmol/mg protein, n = 8) in comparison to I/R-unaffected tissue from the area-not-at-risk (ANAR) (I/R-unaffected: 113.6 ± 44.9 fmol/mg protein, n = 8) of the same hearts (Fig. 4A). Immunoblot analysis also confirmed significantly reduced PDE3A protein levels in cardiac tissue samples that contained infarcted areas (0.78 ± 0.08-fold expression compared to I/R-unaffected, n = 15) compared to unaffected control tissue, whereas PDE1C and PDE2A protein levels were not significantly altered (Fig. 4B and C).

### 3.5 H/R injury impairs human PDE3A protein

**Fig. 5:**
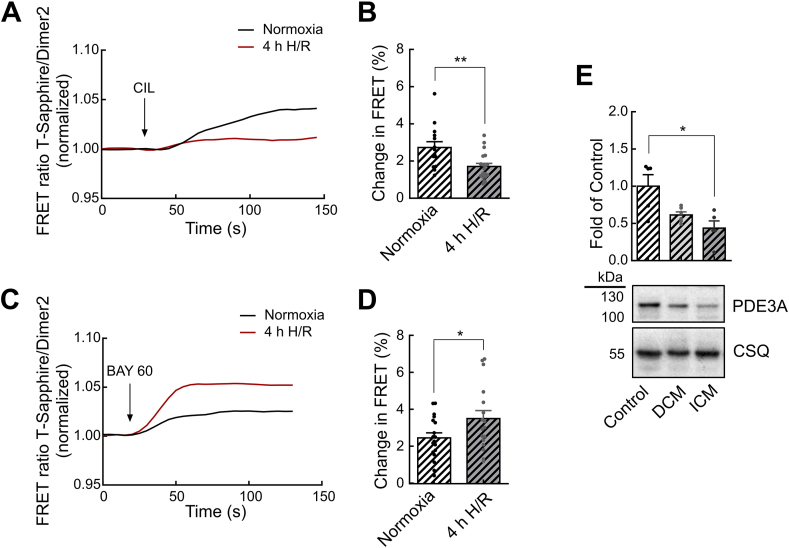
**H/R injury impairs human PDE3A protein. A**-**D** Expression of the red-cGES-DE5 biosensor in human ventricular myocytes was achieved by adenovirus-mediated gene transfer. After 4h H/R or normoxia treatment, cGMP-FRET was measured using the PDE3-selective inhibitor cilostamide (CIL, 10 μM) (**A**, **B**) or the NO-GC activator BAY 60–2770 (BAY 60, 1 μM) (**C**,**D**). Representative FRET traces (**A,C**) and quantification of the FRET responses (**B,D**). Number of measured cardiomyocytes/patients were: normoxia_CIL_ = 14/6, 4h H/R_CIL_ = 19/6; normoxia_BAY 60_ = 18/6, 4h H/R_BAY 60_ = 17/6. **E** Left ventricular tissue samples from patients with dilated (DCM) and ischemic cardiomyopathy (ICM) or non-failing control tissue samples were used for PDE3A immunoblot analysis with calsequestrin (CSQ) as loading control. Representative immunoblots and quantification from n = 5 tissues for control, n = 6 tissues for DCM, and n = 5 tissues for ICM groups are shown. Data in **B** and **D** were analyzed by mixed ANOVA followed by *χ*^2^ test. Significance levels in **E** were tested with Mann-Whitney test. *p < 0.05, **p < 0.01 for normoxia vs 4h H/R. *p < 0.05 for control vs ICM. (For interpretation of the references to colour in this figure legend, the reader is referred to the Web version of this article.)

To compare the results obtained from mouse models with the human setting, we performed cGMP-FRET measurements in isolated human ventricular cardiomyocytes exposed to *in vitro* H/R injury after successful adenovirus-mediated red-cGES-DE5 biosensor expression (Supplementary Fig. S7). To analyze the function of human PDE3 in normoxia versus H/R, FRET-response to CIL was investigated. Compatible with our data from the murine cardiomyocyte H/R model (Fig. 2), PDE3 inhibition raised cGMP levels in normoxia treated human ventricular myocytes (2.7 ± 0.3% change in FRET) but this effect was much weaker after 4h H/R treatment (1.7 ± 0.2%) (Fig. 5A and B). Next, we tested whether the hypoxia-induced reduction of PDE3A function might enhance clinically relevant effects of cGMP-elevating compounds, i.e. the NO-GC activator BAY 60–2770 (BAY 60). Strikingly, the FRET responses to this compound measured in human cardiomyocytes exposed to 4h H/R (3.5 ± 0.4%) were significantly higher than in normoxia treated cells (2.5 ± 0.3%) (Fig. 5C and D).

In addition, we assessed PDE3A protein levels in left ventricular tissue samples obtained from patients with dilated cardiomyopathy (DCM) or ischemic cardiomyopathy (ICM). As controls, non-failing tissue samples were used. PDE3A was significantly reduced in tissue samples from patients with ICM (0.44 ± 0.10-fold of control, n = 5) compared to control (1.00 ± 0.15, n = 5). Interestingly, the decrease in PDE3A expression seemed to be even more pronounced in ICM than in DCM samples (Fig. 5E).

### 3.6 PDE3A is degraded via the autophagosomal-lysosomal pathway during H/R injury

In the mammalian cell, two major systems are responsible for protein degradation, the ubiquitin-proteasome system and autophagy [32]. Autophagy has been shown to be more pronounced in cardiomyocytes exposed to hypoxic or ischemic injury, and cardiomyocytes from HF patients also show increased autophagy [33,34].

To analyze whether PDE3A is degraded via the proteasome during H/R injury, we checked for ubiquitinylated PDE3A protein. Co-immunoprecipitation using antibody against PDE3A with subsequent immunoblotting of mono- and polyubiquitinylated protein conjugates obtained from cardiomyocytes exposed to normoxia or 4h H/R did not reveal significant differences in the overall ubiquitinylation of PDE3A protein (Supplementary Fig. S8A).

**Fig. 6:**
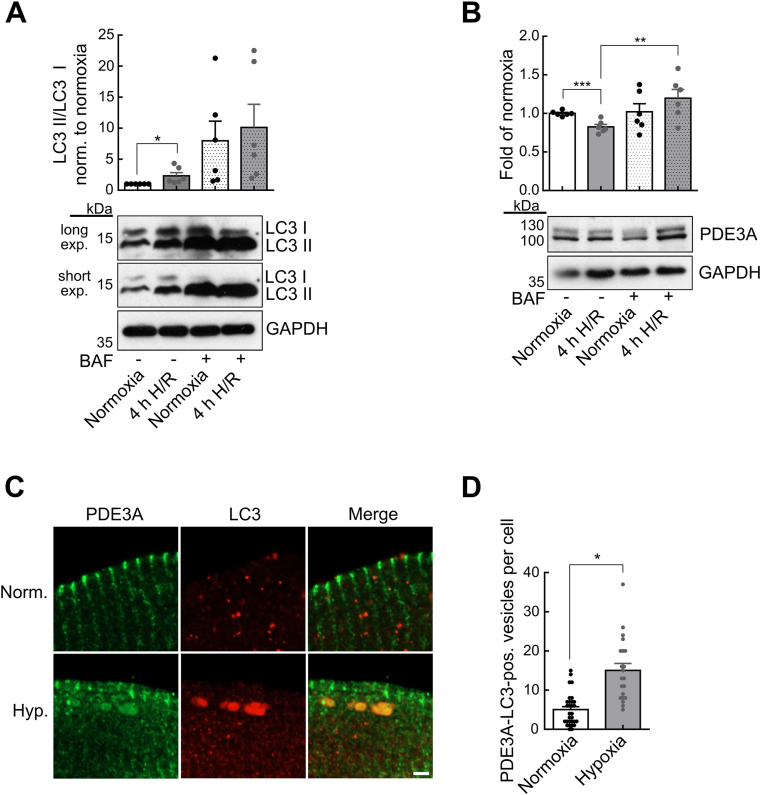
**PDE3A is degraded via the autophagosomal-lysosomal pathway during H/R injury. A** Autophagic flux in normoxia or 4h H/R treated murine cardiomyocytes treated with bafilomycin A1 (BAF, 100 nM) as inhibitor. Representative immunoblots and quantification of LC3 II/LC3 I ratio with GAPDH as loading control (n = 6). **B** Representative immunoblots and quantification of PDE3A protein in murine cardiomyocytes during 4h H/R treatment with/without BAF (100 nM) (n = 6). **C** Representative confocal images of human cardiomyocytes exposed to 4h hypoxia (Hyp.) or normoxia (Norm.) immunostained with anti-PDE3A and anti-LC3. Scale bar, 2 μm. **D** Quantification of PDE3A-LC3-positive vesicles from experiments shown in C. Cardiomyocytes/patients: normoxia = 30/3, hypoxia = 21/3. Data in **A** and **B** were analyzed by unpaired *t*-test, in **D** by mixed ANOVA followed by *χ*^2^ test, *p < 0.05, **p < 0.01, ***p < 0.001.

To monitor autophagy during H/R injury, we determined autophagic flux by evaluating LC3 protein turnover. 4h H/R treated murine cardiomyocytes showed significantly increased LC3 II/LC3 I protein ratios (2.3 ± 0.5-fold, n = 6, compared to normoxia) (Fig. 6A). To further elucidate the mechanism of PDE3A degradation, the vacuolar-type ATPase inhibitor bafilomycin A1 (BAF) was administered to inhibit autophagy. BAF could restore PDE3A protein to physiological levels in 4h H/R treated murine cardiomyocytes (Fig. 6B). To study whether PDE3A is degraded via autophagosomes, human cardiomyocytes exposed to hypoxia or normoxia were immunostained with anti-PDE3A antibody and an antibody against LC3, as a classical marker protein for early autophagosomes [33,34]. We counted PDE3A-LC3 positive vesicles which were in the size range of mammalian autophagosomes (0.5–2 μm) [35]. The number of double positive autophagosomes per cell was significantly increased in cardiomyocytes exposed to hypoxia (15.1 ± 1.8, n = 21) as compared to normoxia treated controls (5.0 ± 0.8, n = 30), indicating that during hypoxic injury, PDE3A is degraded via the autophagy pathway (Fig. 6C and D).

### 3.7 Dose-dependence of PDE3A effect on cardiomyocyte cell death

Previously, it was shown that chronic PDE3 inhibition can promote apoptosis via the so-called PDE3A-cAMP early repressor (ICER) feedback loop [27,36]. This is compatible with detrimental pro-apoptotic and pro-arrhythmic effects of PDE3 inhibitors clinically used in patients with end-stage HF [37,38]. In contrast, cardiomyocyte-specific overexpression of PDE3A1 could reduce the susceptibility to I/R injury, presumably through inhibition of apoptosis [39]. However, here, we could show that increasing basal cGMP levels during H/R injury improves cell survival. Interestingly, the observed downregulation of PDE3A protein by only ∼30–50% during H/R injury increased basal cGMP (Fig. 1F and G) but not cAMP levels in myocytes (Fig. 3E). This led us to the hypothesis that the extent of PDE3A protein reduction determines whether cardioprotection via cGMP or promotion of cell death occurs. Compatible with this hypothesis, we could not detect any upregulation of ICER mRNA during 4h H/R treatment (Supplementary Fig. S8B).

**Fig. 7:**
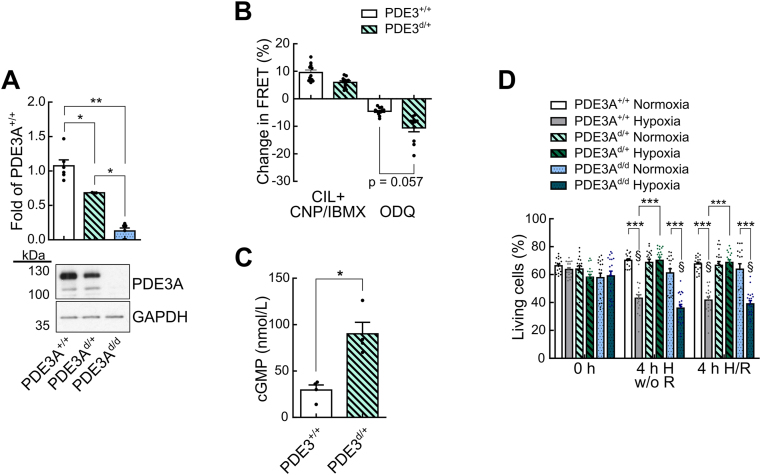
**Dose-dependence of PDE3A effect on cardiomyocyte cell death. A** Representative immunoblots and quantification of PDE3A protein in cardiomyocytes from PDE3A wildtype (PDE3A^+/+^), PDE3A heterozygous (PDE3A^d/+^), and homozygous (PDE3A^d/d^) knockout mice, all expressing Cre recombinase (n = 3–6). **B** PDE3A^+/+^ and PDE3A^d/+^ cardiomyocytes were used for cGMP-FRET 48h after adenovirus-mediated red-cGES-DE5 expression. Maximal cGMP responses were induced by the PDE3 inhibitor CIL (10 μM) followed by saturating concentrations of CNP (1 μM) together with the pan-PDE inhibitor IBMX (100 μM), minimal cGMP levels were achieved by inhibition of NO-GC with ODQ (50 μM). Quantification of FRET signals. Number of cardiomyocytes/mice: PDE3A^+/+^_CIL + CNP/IBMX_ = 11/4; PDE3A^d/+^_CIL + CNP/IBMX_ = 12/4; PDE3A^+/+^_ODQ_ = 12/4; PDE3A^d/+^_ODQ_ = 11/4. **C** Intracellular cGMP concentrations calculated from data in B using a biosensor *in vitro* calibration curve [18] (n = 4). **D** Living cardiomyocyte numbers during hypoxia or normoxia treatment. PDE3A^+/+^, PDE3A^d/+^, and PDE3A^d/d^ cardiomyocytes were exposed to 4h of hypoxia (4h H w/o R) followed by 2h of reoxygenation (4h H/R) or normoxia treatment (n = 3 mice, 6 pictures each). Data in **A** and **C** were analyzed with Mann-Whitney test, data in **B** with mixed ANOVA followed by *χ*^2^ test. Data in **D** were assessed with 2-way ANOVA with Turkey's multiple comparisons test. *p < 0.05, **p < 0.01, ***p < 0.001, §p < 0.001 vs respective t = 0 h group. (For interpretation of the references to colour in this figure legend, the reader is referred to the Web version of this article.)

To prove our hypothesis that the extent of PDE3A protein reduction defines the amount of cardioprotection or cell death, we generated mice with cardiomyocyte-specific deletion of PDE3A using the Cre/loxP system (Supplementary Fig. S9). Immunoblot analysis of PDE3A protein levels in cardiomyocytes from PDE3A wildtype (PDE3A^+/+^Cre^+^) mice, heterozygous conditional knockouts of PDE3A (PDE3A^d/+^Cre^+^), and homozygous conditional knockouts of PDE3A (PDE3A^d/d^Cre^+^) revealed a protein expression of 70% in PDE3A^d/+^Cre^+^ cardiomyocytes compared to PDE3A^+/+^Cre^+^, and as expected, virtually complete absence of PDE3A in PDE3A^d/d^Cre^+^ (Fig. 7A). Measurements of cGMP levels by FRET imaging as described above revealed significantly increased cGMP levels in PDE3A^d/+^Cre^+^ cardiomyocytes (90.1 ± 12.4 nM) compared to PDE3A^+/+^Cre^+^ cardiomyocytes (29.5 ± 5.4 nM) (Fig. 7B and C). The quantification of living and dead cells during H/R treatment in PDE3A^+/+^Cre^+^, PDE3A^d/+^Cre^+^, and PDE3A^d/d^Cre ^+^ cardiomyocytes confirmed that lower PDE3A levels in PDE3A^d/+^ cardiomyocytes protected from cell death during H/R injury, while presence or absence of PDE3A in PDE3A^+/+^Cre^+^ and PDE3A^d/d^Cre^+^, respectively, resulted in significantly increased cell death during H/R treatment compared to normoxic controls (Fig. 7D). Increased basal cGMP levels in PDE3A^d/+^Cre^+^ cardiomyocytes could be a possible explanation for the protection of PDE3A^d/+^Cre^+^ cardiomyocytes from cell death during H/R injury.

## 4 Discussion

Hypoxia is a risk factor in many cardiovascular diseases with serious pathological consequences. It is therefore of great interest to uncover molecular mechanisms that can protect against ischemic and H/R injury. While most of the studies have been focusing on the prevention of apoptosis and the modulation Ca^2+^ homeostasis [40,41], the cGMP signaling pathway has only recently evolved as an attractive therapeutic target. This is emphasized by strong cardioprotective potential of cGMP-elevating drugs in rodent models of I/R injury [14,15,42,43]. Although NO-GC stimulators and neprilysin inhibitors, which increase cardiac cGMP levels, are in clinical use, the regulation of the endogenous cGMP axis during H/R injury in rodent and human cardiomyocytes is largely unknown. Here, we used mouse as well as human myocytes expressing the highly sensitive cGMP-FRET sensor red-cGES-DE5 [5,17] and subjected them to *in vitro* models of H/R injury to study disease relevant changes in intracellular cGMP dynamics.

First, we have demonstrated by live cell imaging and biochemical approaches that basal cGMP levels are significantly increased in mouse cardiomyocytes exposed to H/R. Early studies measuring cGMP levels by biochemical techniques during hypoxic injury have shown contradictory results. While in a Langendorff model of short anoxic injury in rat hearts 10 min anoxia raised cGMP contents [44], hypoxia treatment for 30 min [45] or 40 min with 90 min reoxygenation [46] decreased cGMP contents. Likewise, single cell studies reported inconclusive results. Whereas a 2 h hypoxia treatment could significantly raise cGMP content in adult rat cardiomyocytes in one study [47], neither 30 nor 60 min of anoxia could significantly alter cGMP levels in another study using adult rat cardiomyocytes [48]. In the present work, we could clearly observe a time-dependent increase of cardiomyocyte cGMP levels during H/R and ischemic injury and this increase was already apparent after 90 min of hypoxia and without prior reintroduction of oxygen.

To elucidate the molecular mechanisms which cause this rise in basal cGMP levels, we examined how cGMP is regulated by GCs and PDEs during hypoxic injury. While cardiac deletion of NO-GC did not prevent an increase of cGMP during hypoxia, we could show for the first time that the protein amount of the major cardiac PDE, PDE3A, was significantly reduced in murine cardiomyocytes by the H/R injury. PDE3A depletion was due to protein instability in the absence of any measurable changes in Pde3a (or Gucy*)* mRNA abundance. Interestingly, protein levels of PDE3B, a highly related isoenzyme whose deletion conferred protection against the cardiac I/R injury in mouse [49], remained unchanged during H/R treatment.

Using a FRET-based approach, we could show a significantly reduced cGMP-FRET response to PDE3 inhibition with CIL in myocytes after H/R injury. Furthermore, the PDE3A-dependent cGMP/cAMP crosstalk after H/R injury was significantly less compared to normoxia. This strongly indicates that endogenous PDE3A function is impaired by the H/R injury. Since PDE3A was shown to regulate the degradation of cGMP in adult murine myocytes [5], a reduced function of PDE3A during H/R should cause marked changes in basal cGMP levels which we confirm, to the best of our knowledge, for the first time using our live cell imaging approach.

Acute administration of PDE3 inhibitors has been shown to reduce infarct size when administered before I/R injury in rodents [50,51]. One proposed mechanism to explain how PDE3 inhibition could limit the cardiac damage was the increased opening of mitochondrial Ca^2+^-activated K^+^ channels (mitoK_Ca_) through protein kinase A [51]. More recently, we were able to show that cGMP-elevating compounds reduce cardiac damage through the activation of mitoK_Ca_ channels of the BK type in an open chest model of myocardial infraction [14], although we did not test for a direct link between PDE3-sensititve cGMP pools and mitochondrial BK activity [16]. However, chronic PDE3 inhibition in primary mouse cardiomyocytes has been shown to induce cardiomyocyte apoptosis via a PDE3A-ICER feedback mechanism. The downregulation of PDE3A causes an upregulation of ICER, which in turn represses *Pde3a* gene transcription and promotes apoptosis via downregulation of Bcl-2 protein [27,36]. Here, we showed that increasing basal cGMP levels caused by PDE3A protein downregulation of ∼30–50% during H/R injury improved myocyte survival. This indicates that the extent of PDE3A protein reduction determines whether cardioprotection via the cGMP axis can occur or if, alternatively, apoptosis via cAMP is induced. Accordingly, cardiomyocytes obtained from mice with a heterozygous deletion of one *Pde3a* allele lowering cardiomyocyte PDE3A protein content by ∼30%, increased the basal cGMP levels and protected against H/R-induced cell death. In contrast, cardiomyocytes from *Pde3a* wildtype mice and mice with homozygous deletion of *Pde3a* exhibited lower survival rates under these conditions, supporting our “PDE3A protein dosage” hypothesis. This scenario makes the possible clinical use of low dose PDE3 inhibitor therapy especially attractive for ischemic heart disease and heart failure, which should be further investigated [52].

Although an *in vitro* model of H/R facilitates the investigation of H/R injury [53], its results should be validated under more relevant conditions in intact hearts. Accordingly, two pathophysiological relevant settings, a Langendorff model of global anoxia and an acute open chest in situ model of I/R injury, were investigated to cover the whole complexity of ischemic injury. In both models, we could confirm that cGMP levels increased after the hypoxic insult. The *in vivo* mouse model also demonstrated that this increase is spatially confined to myocardium at risk and is accompanied by a depletion of the PDE3A protein. In line with these findings, we previously identified by optical means using the red-cGES-DE5 sensor mice a cinaciguat-induced increase in cardiac cGMP in situ [14]. Furthermore, we could demonstrate that the cardiomyocyte-specific ablation of NO-GC in mice does not per se affect the vulnerability to I/R injuries [15], indicating that in this model the CNP-stimulated GC as well as PDE3A determine basal cGMP dynamics, while NO-GC could be important for mechanical post-conditioning procedures and the cardioprotection afforded by cGMP-elevating compounds such as NO-GC stimulators. Collectively, these data suggest that in the ischemic myocardium, endogenously triggered mechanisms could act together with pharmacological approaches in order to stimulate the cGMP axis and thereby the cardioprotective potential of this cascade.

PDE3A is the most abundant PDE family expressed in human cardiomyocytes [54]. Here, we have for the first time established FRET-based cGMP measurements in human ventricular myocytes. We could successfully confirm the effects of H/R injury on PDE3A activity as deduced from i.) cGMP-FRET responses recorded in the presence of the selective inhibitor CIL and ii.) significantly reduced PDE3A protein levels in ventricular tissue biopsies derived from ICM patients. Interestingly, an even more pronounced decrease in PDE3A protein was seen in ICM-derived samples than in the case of DCM. Importantly, this reduced PDE3 activity significantly enhanced the effects of the NO-GC activator BAY 60–2770 on cyclase activity, which might have immediate translational implications on the clinical use of NO-GC stimulators/activators.

In human cells, we could also uncover the mechanism leading to PDE3A protein instability in cardiomyocytes. When exposed to H/R injury, these cells show an intensive co-localization of PDE3A with the autophagosome marker LC3, suggesting that the autophagy pathway is used by the cell to downregulate PDE3A protein levels, and thereby the ability of this enzyme to degrade cGMP. Autophagy is known to be more active in cardiomyocytes exposed to hypoxic or ischemic injury and in failing hearts [33,34] and autophagic flux was significantly increased in our H/R model. Importantly, inhibition of autophagy with bafilomycin A1 could restore PDE3A protein to physiological levels in 4h H/R treated murine cardiomyocytes. Although the mechanism underlying autophagosome biogenesis is not completely understood, there is some consensus that autophagosomes are formed from preexisting membrane compartments called omegasomes. Omegasomes can originate from endo/sarcoplasmatic reticulum (ER/SR) [55,56], which has been described as the major PDE3A localization site [49,57]. It is tempting to speculate that ER/SR stress occurring during hypoxia leads to formation of autophagosomes, which in turn include parts of ER/SR membranes containing PDE3A. Recently, the PDE5 inhibitor icariside II was shown to exert beneficial effects in a rat model of cerebral I/R-induced injury via reducing the autophagic flux. The authors suggested a crosstalk between cGMP-dependent protein kinase I (cGKI *aka* PKG), glycogen synthase kinase-3β (GSK-3β) and autophagy as possible mechanism [58]. The role of PKG and GSK-3β in the regulation of autophagy in our H/R model remains unclear. However, it appears that the PDE3A degradation during H/R injury serves as an autoregulatory protective mechanism that ultimately improves the cardioprotective potential of cGMP. cAMP has also been described as potent inducer of autophagy, providing a potential feed-forward control mechanism over PDE3A protein levels in H/R exposed cardiomyocytes. However, hypoxia did not lead to significant changes in basal cAMP concentrations ruling out unexpected effects of cAMP on autophagic flux and thus PDE3A degradation and apoptosis in adult cardiomyocytes.

## 5 Conclusions

Collectively, our mouse and human data provide a new regulatory mechanism, which activates the cGMP signaling cascade during H/R injury and enhances cGMP responses to pharmacological NO-GC activation. This mechanism might represent a promising target for the treatment of ischemic heart disease and ischemic cardiomyopathies. In the future, it would be important to study whether patients with such hypoxia/ischemia-associated types of cardiac pathology may profit even more from cGMP-based therapies.

## Funding

This work was supported by the Deutsche Forschungsgemeinschaft Research Unit 2060 “cGMP Signaling in Cell Growth and Survival” (grant FOR2060 to VON, RL, and AF) and the Gertraud und Heinz-Rose Stiftung (grant to VON). RL and MCS are members of the 10.13039/501100001659Deutsche Forschungsgemeinschaft Research Training Group “cGMP: From Bedside to Bench” (grant number 335549539/GRK 2381).

## Author contribution

VON and RL designed the project. NIB, AK, and MCS conducted experiments and analyzed the data. AF provided NO-GC KO mice and antibodies. BR, HR and CEM provided human ventricular tissue samples, CEM provided isolation protocols and patient data. BVS, MK and TSR generated the conditional Pde3a KO mouse model. NIB, RL, and VON wrote the manuscript. All authors edited and approved the manuscript for submission.

## Declaration of competing interest

RL has a cooperation with Cyclerion Therapeutics Inc. on a topic unrelated to this study. All other authors have stated explicitly that there are no conflicts of interest in connection with this article.
