# Supplementary material for: Rise of cGMP by partial phosphodiesterase-3A degradation enhances cardioprotection during hypoxia
Source: Redox Biol. 2021 Nov 6;48:102179. doi: 10.1016/j.redox.2021.102179 (PMC8590074; doi:10.1016/j.redox.2021.102179)
Supplement: Multimedia component 1 [file mmc1.pdf]

Supplementary Material

Rise of cGMP by partial phosphodiesterase-3A degradation enhances cardioprotection during hypoxia

Nadja I. Bork[a,b], Anna Kuret[c], Melanie Cruz Santos[c], Cristina E. Molina[a,b], Beate Reiter[d], Hermann Reichenspurner[d], Andreas Friebe[e], Boris V. Skryabin[f], Timofey S. Rozhdestvensky[f], Michaela Kuhn[e], Robert Lukowski[c], Viacheslav O. Nikolaev[a,b,*]

[a] Institute of Experimental Cardiovascular Research, University Medical Center Hamburg-Eppendorf, Hamburg, Germany
[b] DZHK (German Center for Cardiovascular Research), partner site Hamburg/Kiel/Lübeck, Hamburg, Germany
[c] Department of Pharmacology, Toxicology and Clinical Pharmacy, Institute of Pharmacy, University of Tübingen, Tübingen, Germany
[d] Department of Cardiovascular Surgery, University Heart & Vascular Center Hamburg, Hamburg, Germany
[e] Physiologisches Institut, University of Würzburg, Würzburg, Germany
[f] Core Facility Transgenic Animal and genetic engineering Models (TRAM), University of Münster, Münster, Germany

*Address for correspondence:

Viacheslav O. Nikolaev
Institute of Experimental Cardiovascular Research
University Medical Center Hamburg-Eppendorf (UKE)
Martinistr. 52
D-20246 Hamburg, Germany
Phone: +49 40 7410 51391; Fax: +49 40 7410 40180
Email: v.nikolaev@uke.de

SUPPLEMENTARY METHODS

Generation of Pde3a conditional KO mouse model.

Cytoplasmic microinjections of the CRISPR-Cas9 components and Pde3a template into fertilized mouse oocytes. Commercially synthesized crRNAs (Pde3A_crRNA2: CCATGCTTCTTACCAAAGCC; Pde3A_crRNA4: CTCTAAAAATTCTGATGACT), tracrRNA and Cas9 protein [Integrated DNA Technologies (IDT), USA] were mixed at following conditions: 100 pmol of each crRNA was mixed with 200 pmol of tracrRNA in 10 mM potassium acetate, 3 mM HEPES (pH 7.5) buffer and, incubated at 95°C for 2 min followed by cooling to room temperature. The annealed crRNA/tracrRNA complexes were mixed with Cas9 protein, Cas9 mRNA, and synthesized Pde3a template DNA fragment (Supplementary Fig. S9A) (Biomatik, USA) as described previously [1]. The microinjection solution containing: 2 pmol/µL of each crRNA, 4 pmol/µL of tracrRNA, 25 ng/µL of Cas9 protein, 10 ng/µL of Cas9 mRNA, and 0.05 pmol/µL of DNA template was filtered through 0.22 µm centrifugal columns (Millipore). Cytoplasmic microinjections were performed into B6D2F1 (C57BL/6 x DBA) zygotes in M2 media using the Diaphot 300 inverted microscope, Transjector 5246 (Eppendorf), and Narishige NT-88NE micromanipulators. After microinjections, the two-cells embryos were surgically transferred to oviducts of pseudopregnant CD1 foster mice and carried to term.

qPCR analysis of the targeting events and sequencing of the Pde3a exon 12 genomic locus. TaqMan qPCR analysis was performed in 20 µL volume using LightCycler 480 system (Roche). For detection of the 5' and 3' correctly targeted regions we designed assays with external primers (PDE_d1 and PDE_r1, respectively), located outside of the targeting homology and internal primers LoxA1rev and LoxA2dir, respectively. TaqMan UPD probe #46 (Roche, cat.no. 04688066001) was used in both assays. Hence, 5' assay was performed with PDE_d1 (CATCATTACAGATCATAACAGAATCCA), LoxA1rev (GCCCTCAAGTAGGTTGTTTCC) and TaqMan UPD probe: #46 (Roche); whereby for 3' assay the following primers were used: PDE_r1 (CGAAGGCAACTGAGAAAAGACC), LoxA2dir: (CCAGGTTAGGATCGCATAGG) and TaqMan UPD probe: #46. The correct size (of 219 bp and 174 bp for 5' and 3' assays, respectively) of

resulting qPCR amplicons were confirmed using 6% (w/v) polyacrylamide gel (1 x TBE buffer) electrophoresis followed by ethidium bromide staining and sequencing. Pde3a targeted genomic locus was sequenced using pairs: LoxA1dir (AAGCTGGGCCCTGGTAAT) / PD3_qPCRr2 (CTGGGTGATCGTAGTCATGC) for 5'- part and PD3_qPCRd2(CAAATATGGATGCCTGTCTGG) / LoxA2rev (CGACTACGCGCATTAAGGA) for 3'- part.

Southern blot DNA analysis. Genomic DNA was isolated using mouse tail biopsies. Tail tissue was lysed overnight at 55°C in 100 mM Tris-HCl (pH 8.5), 5 mM EDTA, 0.2% SDS, 200 mM NaCl, and proteinase K (100 µg/mL) (Roche) buffer. Genomic DNA was extracted by phenol-chloroform/chloroform method, precipitated by 2.5 volume of 96% ethanol and washed with 70% ethanol. Approximately 10 to 20 µg of TE dissolved (10 mM Tris, pH 7.9; and 0.2 mM EDTA) genomic DNA was digested with restriction endonuclease (*Hind*III or *Bam*HI), fractionated on 0.8% agarose gels, and transferred to GeneScreen nylon membranes (NEN DuPont). The membranes were hybridized with ^{32}P-labeled Pde3a donor DNA fragment, used as a specific probe (Supplementary Fig. S9A,E,F). Membranes were washed with SSPE buffer (0.09 M NaCl, 5 mM NaH_2PO_4, and 0.5 mM EDTA [pH 7.7]) and 0.5% sodium dodecyl sulfate at 65°C and exposed to MS-film (Kodak) at -80°C (Supplementary Fig. S9E,F).

Clinical patient characteristics. All human ventricular tissue samples were obtained from patients with/without HF at the time of surgery. Tissues used for myocyte isolation, FRET-recordings, and immunostaining were obtained from 16 patients (9 men, 7 women, mean age 48 ± 4 years) with normal left ventricular ejection fraction (mean LVEF: 58 ± 4%).

Left ventricular tissue samples for immunoblot analysis was obtained from 6 DCM patients (5 men, 1 woman; mean age 54 ± 4 years) with a mean LVEF of 21 ± 2% and 5 ICM patients (4 men; 1 woman; mean age 64 ± 3 years) with a mean LVEF of 25 ± 2%. Control tissue samples were obtained from 5 patients (3 men, 2 women; mean age 45 ± 13 years) with normal LVEF (mean: 60 ± 2%).

Adenoviral transduction of human and murine ventricular myocytes. To express the red-cGES-DE5 biosensor in human (Fig. 5) or murine (Fig. 7) ventricular myocytes, we used our previously developed biosensor expression adenovirus [2] applied at a multiplicity of infection 300. Cells were cultured at 37°C and 5% CO_2 for 48 h prior to measurements to enable proper biosensor expression.

Förster resonance energy transfer (FRET) imaging

Single cell FRET measurements. Single cell FRET measurements were performed as described previously [3]. The biosensors red-cGES-DE5 [4] or Epac1-camps [5] were excited at 400 nm or 440 nm, respectively, using pE-100 CoolLED Illumination System. A Dual View beamsplitter (DV2, Photometrics) (Cube 11-EM, 565 dcxr, 520/30, D630/50 for red-cGES-DE5; Cube 05-EM, 505 dcxr, D480/30, D535/40 for Epac1-camps) was used to split the emission light into donor and acceptor channels. Recordings were done with an OptiMOS CMOS camera (QImaging). Compounds were applied at room temperature (RT) as bath applications, images were taken every 5 seconds. Bleedthrough factor correction and data analysis were performed exactly as described [3].

Whole heart FRET measurements. cGMP-FRET in Langendorff hearts was monitored with a stereomicroscope (M165F, Leica) as described previously [6]. The red-cGES-DE5 biosensor was excited with a 400 nm LED (pE-100, CoolLED). Emission light was split into donor and acceptor channels with a beamsplitter (Cube 11-EM, 565 dcxr, D520/30, D630/50) and recorded with an OptiMOS CMOS camera (QImaging). Images were taken every 30 seconds. Micro Manager 1.4.5 software was used for time-laps image acquisition. Recording was started after 20 min of equilibration.

FRET measurements of heart lysates in buffers with different pH. Heart lysates from transgenic red-cGES-DE5 [4] and Epac1-camps [5] mice on C57BL6/J background were prepared in Tris/EDTA buffer (Tris: 5 mM; EDTA 2 mM, pH 7.4) using and Ultra Turrax (T-10 basic, IKA). Lysates were diluted in Tris/EDTA buffer of different pH. Fluorescence of donor and acceptor

fluorophores was detected in a FlexStation 3 plate reader (Molecular Devices) and analyzed with Soft Max Pro Software (Molecular Devices).

Western blot analysis. To prepare protein lysates from mouse ventricular cardiomyocytes exposed to H/R injury, cells were washed with ice cold phosphate-buffered saline (PBS), harvested (3000 rpm, 3 min, 4°C), and resuspended in homogenization buffer (in mM): EGTA 1, HEPES 10, NaCl 150, sucrose 300, triton 1%, phosphatase and protease inhibitors (Roche). Cells were lysed using an insulin syringe (Omnican, Braun). To prepare protein lysates from mouse hearts, snap frozen tissues were homogenized three times for 20 s in homogenization buffer with an Ultra Turrax (T-10 basic, IKA). Human ventricular tissue samples were homogenized four times 30 s in RIPA buffer containing NaCl (150 mM), triton (1%), SDS (0.1%), sodium deoxycholate (0.5%), tris (pH 8.0, 50 mM), phosphatase and protease inhibitors (Roche). Protein lysates were centrifuged (13000 rpm, 10 min, 4°C) and supernatants were used to determine protein concentrations using the Pierce BCA protein assay kit (Thermo Scientific). Protein lysates were mixed with 3 x SDS Stop (Tris, 200 mM, pH 6.7; SDS, 6%; glycerin, 15%; bromphenolblue; β-mercaptoethanol, 10%), denatured for 10 min at 70°C, and stored at -20°C.

Proteins were size-separated using SDS polyacrylamide gel electrophoresis (SDS-PAGE). 10-30 µg protein were loaded on 8-15% polyacrylamide gels. Protein Marker V (Peqlab) or Precision Plus Protein Dual Color (Biorad) was used as protein size standard. Proteins were transferred on nitrocellulose or PVDF membrane (Amersham and Biorad) with tank blot method.

For immunodetection, primary Calsequestrin (1:5000 dilution, Thermo Scientific #PA1-913), GAPDH (1:160000 dilution, Bio Trend #5G4), GC-B (1:2500 dilution, generous gift from H. Schmidt), HIF-1α (1:1000 dilution, Thermo Scientific #PA1-16601), LC3 (1:1000 dilution, Cell Signaling #3868), Mono- and polyubiquitinylated conjugates (1:50000 dilution, Biomol #BML-PW8810), NO-GCα_1 (1:2000 dilution, self-made), NO-GCβ_1 (1:2000 dilution, self-made), PDE1C (1:1000 dilution, generous gift from C. Yan), PDE2A (1:500 dilution, Fabgennix #PD2A-101AP), PDE3A (1:1000 dilution, generous gift from C. Yan), PDE3B (1:2000 dilution, generous gift from S. Rybalkin), PDE4B (1:2500 dilution, abcam #ab170939), and PDE4D (1:2500 dilution, abcam

#ab171750) antibodies were used. Horseradish peroxidase (HRP)-conjugated secondary antibodies anti-mouse (Biorad #170-5047), anti-rabbit (Biorad #170-5046), and anti-guinea pig (Merck #AP108P) in 1:5000 dilutions were used for detection with the Super Signal West Pico PLUS kit (Thermo Fisher). X-ray films (Fujifilm) were developed in a medical film processor (SRX-101A, Konica Minolta) and analyzed using ImageJ software.

Co-Immunoprecipitation. For immunoprecipitation (IP), murine cardiomyocytes exposed to normoxia or 4h H/R were lysed in IP buffer (PBS containing protease inhibitor (Roche, according to the manufacturer's instructions) and triton (1%)) using an insulin syringe (Omnican, Braun). Lysates were incubated with antibody and 20 µL of A/G plus agarose beads (Santa Cruz) and incubated overnight using a rocking shaker at 4°C. Beads were washed twice with PBS and once with IP buffer and elution was performed at 95°C for 5 min in 3 x SDS Stop. Eluted proteins were size-separated on SDS-PAGE gels and developed by immunoblotting as described above.

Cycloheximide protein chase assay. Protein synthesis inhibitor cycloheximide (CHX) was used to investigate protein stability in cardiomyocytes exposed to H/R injury. Therefore, CHX (50 µM) was added onto the plated myocytes before H/R treatment. After H/R, cells were harvested, and protein lysates were prepared for Western blot analysis.

Lactate dehydrogenase assay. LDH release from cardiomyocytes into the medium was quantified after normoxia or 4h H/R treatment by using the LDH-Glo Cytotoxicity Assay kit according to manufacturer's protocol (Promega).

Quantitative real time polymerase chain reaction. Total RNA from murine ventricular cardiomyocytes was isolated using the RNeasy fibrous tissue mini kit (Qiagen). mRNA was transcribed into cDNA using the iScript cDNA synthesis kit (BioRad) with 200 ng of RNA per reaction. Rotor-Gene SYBR Green PCR Kit (Qiagen) was used for quantitative real-time PCR reaction (qRT PCR) in a Rotor Gene Q cycler (Qiagen). Samples were run in triplicates using the

following conditions: 10 min at 95°C, followed by 40 x three step cycling (10 s at 95°C, 15 s at 60°C, 20 s at 72°C), and running a melting curve (72 – 95°C, 0.5°C steps). GAPDH was used to normalize target genes.

The following primer pairs were used: *Gapdh* (forward: 5'-CGTCCCGTAGACAAAATGGT -3'; reverse: 5'-GAATTTGCCGTGAGTGGAGT -3'), *Gucy1a1* (forward: 5'-CCCCTGGTCAGGTTCCTAAG-3'; reverse: 5'-GGAGACTCCCTTCTGCATTCT-3'), *Gucy1b1* (forward: 5'-CTGCTGGTGATCCGCAATTAT-3'; reverse: 5'-GATGGTATCATAGCCAGACTCCT-3'), *Icer* (forward: 5'- TTGCCCCAAGTCACATGGC-3'; reverse: 5'-ACTGCGACTCGACTCTCAAGA-3'); *Npr1* (forward: 5'-TGGAGACACAGTCAACACAGC-3'; reverse: 5'-CCGAAGACAAGTGGATCCTG-3'), *Npr2* (forward: 5'-TGTTTGGTGTTTCCAGTTTCC-3'; reverse: 5'-AGTTCTTCCCAGCGAATGC-3'), *Pde3a* (forward: 5'-TGTTTGAAGACATGGGGCTCT-3'; reverse: 5'-TAGAACATCGGTGGCATGGATT-3').

Immunostaining. For immunostaining of hypoxia or normoxia treated human ventricular cardiomyocytes, cells were washed once with PBS and fixed by incubation with ice-cold ethanol (99%) for 20 min at -20°C. After washing three times with PBS, cells were incubated in blocking buffer (PBS containing triton (0.3%), and FCS (10%)) for 2h at RT. Anti-PDE3A (1:100 dilution, Santa Cruz #sc 11832), and anti-LC3 (1:100 dilution, Cell Signaling #3868) were used as primary antibodies. As secondary antibodies Alexa Fluor 488 donkey anti-goat (Thermo Scientific #A11055) and Alexa Fluor 568 donkey anti-rabbit (Thermo Scientific #A10042) were used.

Confocal microscopy. Confocal pictures were taken at a Zeiss LSM 710 NLO microscope using a Plan-Apochromat 63x/1.40 oil-immersion objective with 488 nm and 561 nm laser excitation. ZEN 2010 software (Zeiss) was used for image analysis.

cGMP immunoassay. cGMP levels in mouse ventricular cardiomyocytes or heart tissue samples were quantified with a cGMP immunoassay kit (Sigma Aldrich). Samples were homogenized in HCl (0.1 M) using an insulin syringe (Omnican, Braun) for isolated cardiomyocytes or an Ultra Turrax

(T-10 basic, IKA) for tissue samples. Pierce Coomassie protein assay kit (Thermo Scientific) was used to measure protein content.

SUPPLEMENTARY FIGURES

Fig. S1

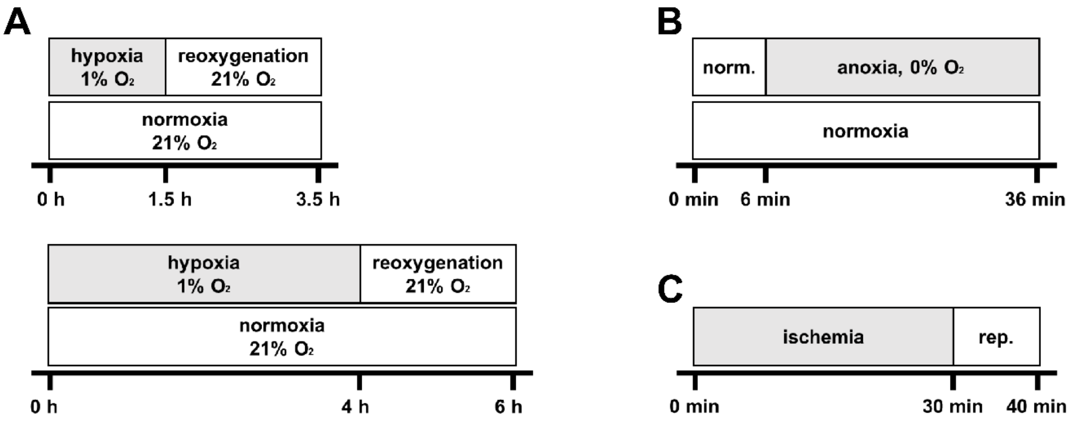

Supplementary Fig S1. Models of hypoxia/reoxygenation and ischemia/reperfusion injury. **A** *In vitro* hypoxia/reoxygenation (H/R) injury in myocytes was created in a modular incubator chamber flushed with N_2 and CO_2. The O_2 concentration in the chamber was 1% (94% N_2, 5% CO_2, and 1% O_2). Hypoxia duration was 90 min or 4h, reoxygenation duration was 2h under atmospheric oxygen at 37°C and 5% CO_2. **B** Langendorff whole heart model of global anoxic injury. Langendorff hearts were perfused for 6 min with carbogen aerated (95% O_2, and 5% CO_2) modified Krebs-Henseleit solution. Afterwards, non-oxygenated solution was perfused for 30 min to induce global anoxic injury. Perfusion was carried out retrogradely under constant pressure (80 mmHg) and permanent pacing at 530 beats per minute. **C** Open chest model of ischemia/reperfusion (I/R) injury. Left coronary artery was occluded for 30 min with subsequent reperfusion for 10 min.

Fig. S2

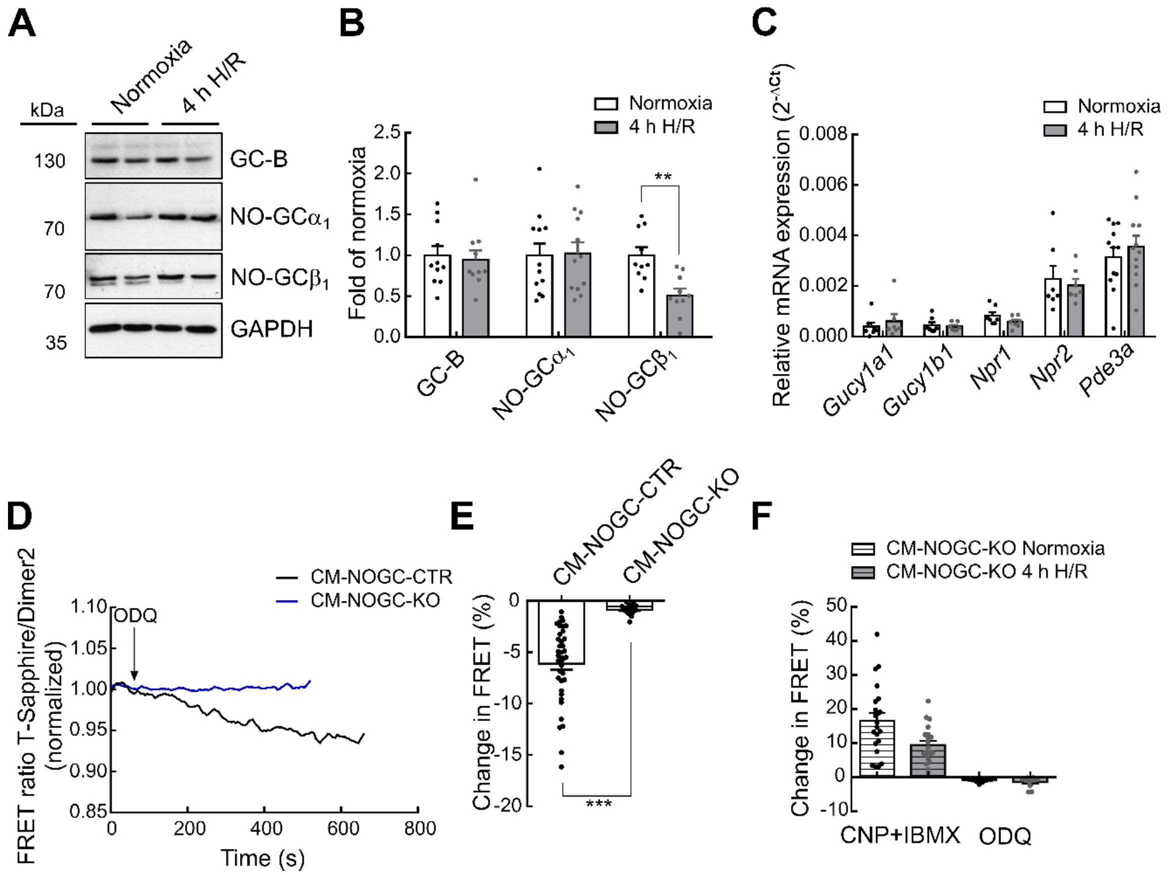

Supplementary Fig S2. cGMP generating guanylyl cyclases (GCs) after *in vitro* H/R injury and functional analysis of NO-GC knockout cardiomyocytes. **A**, **B** Representative immunoblots (**A**) and quantification (**B**) of GC-B, NO-GCα_1, and NO-GCβ_1 proteins in cardiomyocytes after 4h H/R. Protein levels were normalized to GAPDH (n=10-12 mice per condition). **C** Relative gene expression of *Gucy1a1*, *Gucy1b1*, *Npr1*, *Npr2*, and *Pde3a* genes in myocytes after 4h H/R or normoxia normalized to *Gapdh* (n=7 mice for *Gucy1a1*, *Gucy1b1*, *Npr1*, and *Npr2*, n=12 mice for *Pde3a*). **D**, **E** Representative cGMP-FRET traces (**D**) and quantification (**E**) from control (CM-NOGC-CTR) and cardiomyocyte-specific NO-GC knockout (CM-NOGC-KO) red-cGES-DE5 myocytes upon NO-GC inhibition with ODQ (50 µM). Number of cardiomyocytes/mice were: CM-NOGC-CTR=39/9, CM-NOGC-KO=16/3. **F** Quantified maximal/minimal cGMP-FRET responses of CM-NOGC-KO cardiomyocytes exposed to normoxia or 4h H/R. Maximal cGMP contents were reached by using saturating concentrations of natriuretic peptide CNP (1 µM) together with the pan-PDE inhibitor (IBMX, 100 µM), minimal cGMP contents by NO-GC inhibition with ODQ (50 µM). Number of cardiomyocytes/mice were: CM-NOGC-KO normoxia$_{CNP+IBMX}$=22/3; CM-NOGC-KO 4h H/R$_{CNP+IBMX}$=21/3; CM-NOGC-KO normoxia$_{ODQ}$=16/3; CM-NOGC-KO 4h H/R$_{ODQ}$=12/3. Data in **B** and **C** were analyzed by Mann-Whitney test. Significance levels in **E** and **F** were tested with mixed ANOVA followed by χ^2 test. ** p<0.01 for normoxia vs 4h H/R in B, *** p<0.001 for NO-GC CTR vs NO-GC KO in E.

Fig. S3

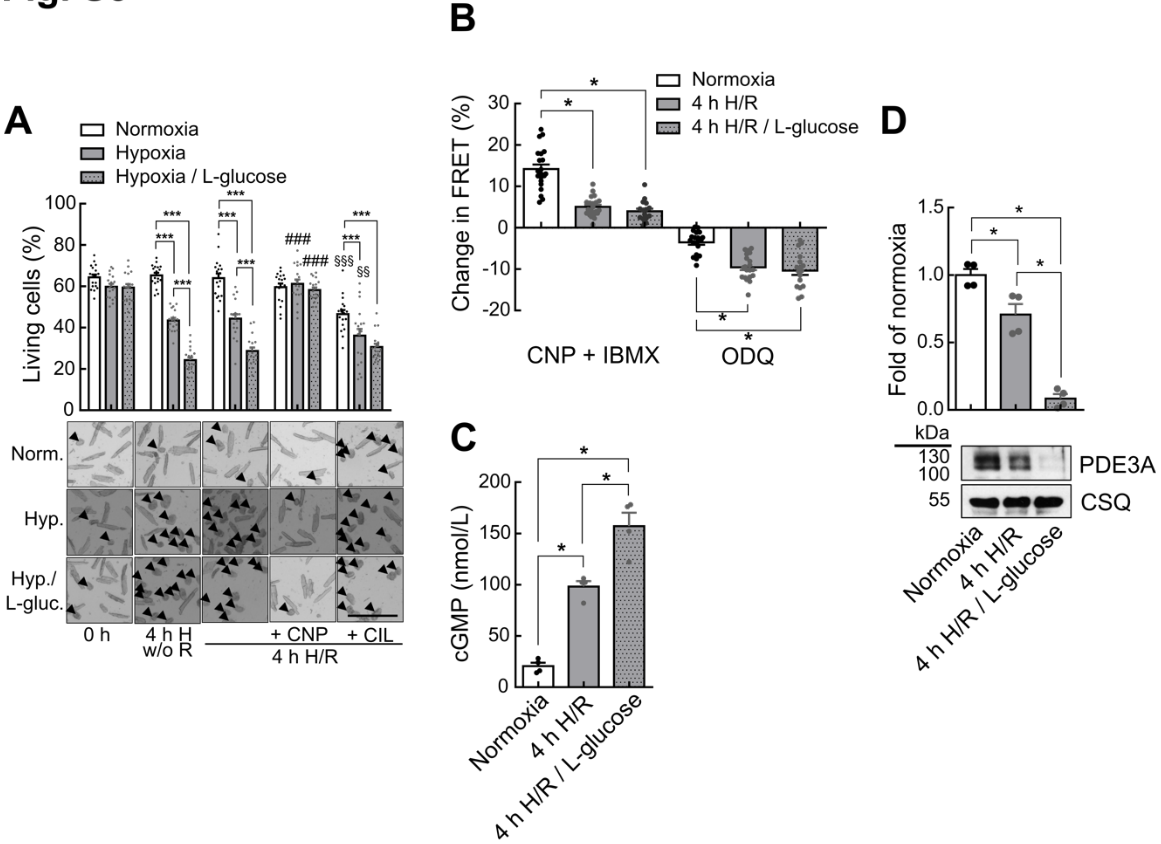

Supplementary Fig S3. *In vitro* H/R injury with concomitant glucose deprivation increases basal cGMP levels more drastically than hypoxia/reoxygenation injury in murine ventricular myocytes. A Light microscopy images and quantification of cardiomyocytes taken before induction of hypoxia (0h), 4h after hypoxia without reoxygenation (4h H w/o R) and 2h after reoxygenation (4h H/R) with (hyp./L-gluc.) or without (hyp.) concomitant glucose deprivation. Some cells were treated with CNP (100 nM) or CIL (10 µM) during hypoxic treatment. Normoxic treated cells were used as controls. Black arrowheads indicate dead cardiomyocytes. Scale bar, 250 µm (n=3 mice, 6 pictures each). **B** Quantification of FRET measurements in red-cGES-DE5 cardiomyocytes after normoxia, 4h H/R or 4h H/R with concomitant glucose deprivation (4h H/R / L-glucose). Saturating concentrations of CNP (1 µM) together with the pan-PDE inhibitor IBMX (100 µM) were used to measure maximal cGMP contents, minimal cGMP levels were reached with the NO-GC inhibitor ODQ (50 µM). Number of measured cardiomyocytes/mice were: normoxia$_{CNP+IBMX}$=21/4; 4hH/R$_{CNP+IBMX}$=21/4; 4hH/R/L-glucose$_{CNP+IBMX}$=16/4; normoxia$_{ODQ}$=20/4; 4hH/R$_{ODQ}$=20/4; 4hH/R/L-glucose$_{ODQ}$=17/4. **C** Intracellular cGMP concentrations calculated from data in B using a biosensor *in vitro* calibration curve [7] (n=4 mice). **D** Representative immunoblots and quantification of PDE3A protein in cardiomyocytes after hypoxia/L-glucose / hypoxia / normoxia treatment with calsequestrin (CSQ) as loading control (n=4 mice). Data in **A** were analyzed by 2-way ANOVA with Turkey's multiple comparisons test, in **B** by mixed ANOVA followed by χ^2 test, data in **C** and **D** by Mann-Whitney test, * p<0.05, ** p<0.01, *** p<0.001 as indicated, ### p<0.001 and §§ p<0.01, §§§ p<0.001 vs respective untreated 4h H/R group.

Fig. S4

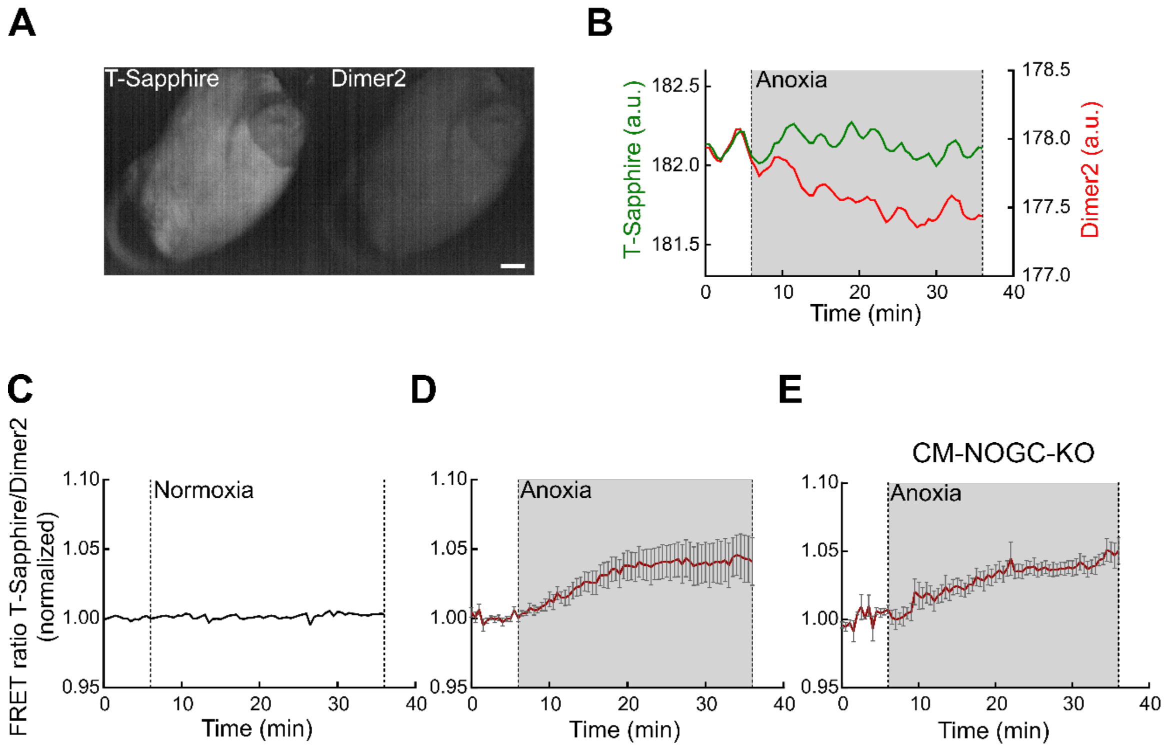

Supplementary Fig S4. Global anoxic injury in Langendorff hearts raises cGMP content. To monitor cGMP levels during global anoxic injury, anoxic red-cGES-DE5 Langendorff hearts were used. **A** T-Sapphire and Dimer2 fluorescence in a Langendorff heart during cGMP-FRET measurements. Scale bar, 1 mm. **B** Exemplary FRET trace, showing fluorescence intensities of the two fluorophores of the red-cGES-DE5 biosensor during global anoxic injury. **C** Normalized representative cGMP-FRET recording of a normoxia treated control. **D**,**E** Averaged cGMP-FRET ratios during global anoxic injury in (**D**) red-cGES-DE5 hearts (n=7), and in (**E**) cardiomyocyte-specific NO-GC knockout (CM-NOGC-KO) / red-cGES-DE5 hearts (n=4).

Fig. S5

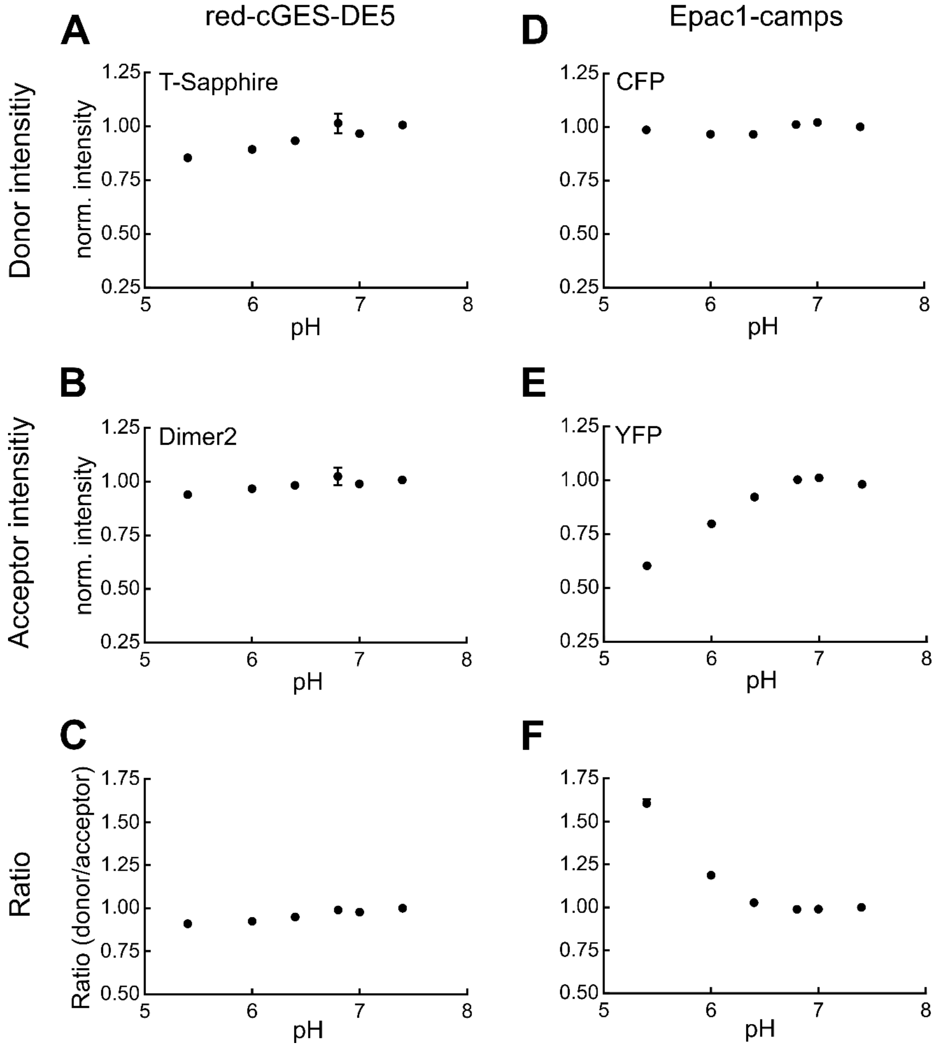

Supplementary Fig S5. pH sensitivity of donor and acceptor fluorophores in red-cGES-DE5 and Epac1-camps FRET biosensors. Normalized fluorescence intensity of donor and acceptor fluorophores and their ratio for the cGMP-based FRET sensor red-cGES-DE5 (**A**,**B**,**C**) and the cAMP-based sensor Epac1-camps (**D**,**E**,**F**) measured *in vitro* at different pH values. Heart lysates of n=5 were measured per condition. Lowering pH does not affect the performance of the red-cGES-DE5 biosensor, whereas YFP intensity of Epac1-camps is dampened at pH<6.

Fig. S6

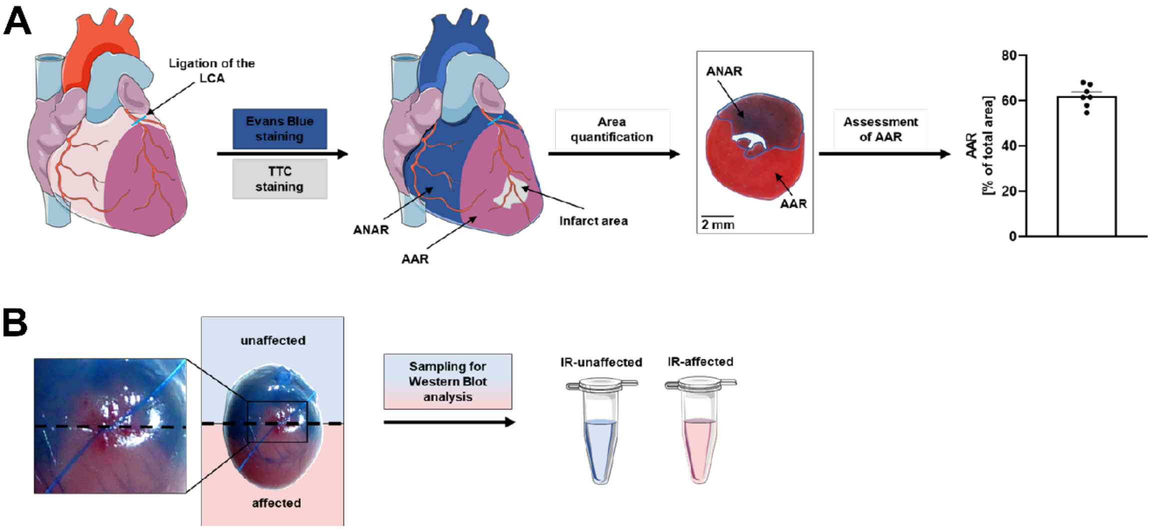

Supplementary Figure S6. Workflow to identify I/R-affected/unaffected myocardium in an acute open chest in situ model of I/R injury. A Retrograde injection of 1% Evans Blue solution to distinguish cardiac tissue affected from left coronary artery (LCA) occlusion. Area-at-risk (AAR) is defined as percentage of total heart area (n=7). **B** I/R-exposed hearts (30 min of ischemia following 10 min of reperfusion) were divided into "I/R-affected" myocardium (AAR, red in (A) and (B) plus a minor fraction of "contaminating" cells from the area-not-at-risk (ANAR) in the right ventricle) and "I/R-unaffected" myocardium (blue in (A) and (B)) from the ANAR. Atria and vessels (not shown) were removed prior to subsequent protein purification steps. The localization of the LCA solution ((B), left panels) allowed a clear distinction between "I/R-affected" and "I/R-unaffected" cardiac muscle. *Heart illustrations in (A) were modified from Servier Medical Art images (smart.sevier.com).*

Fig. S7

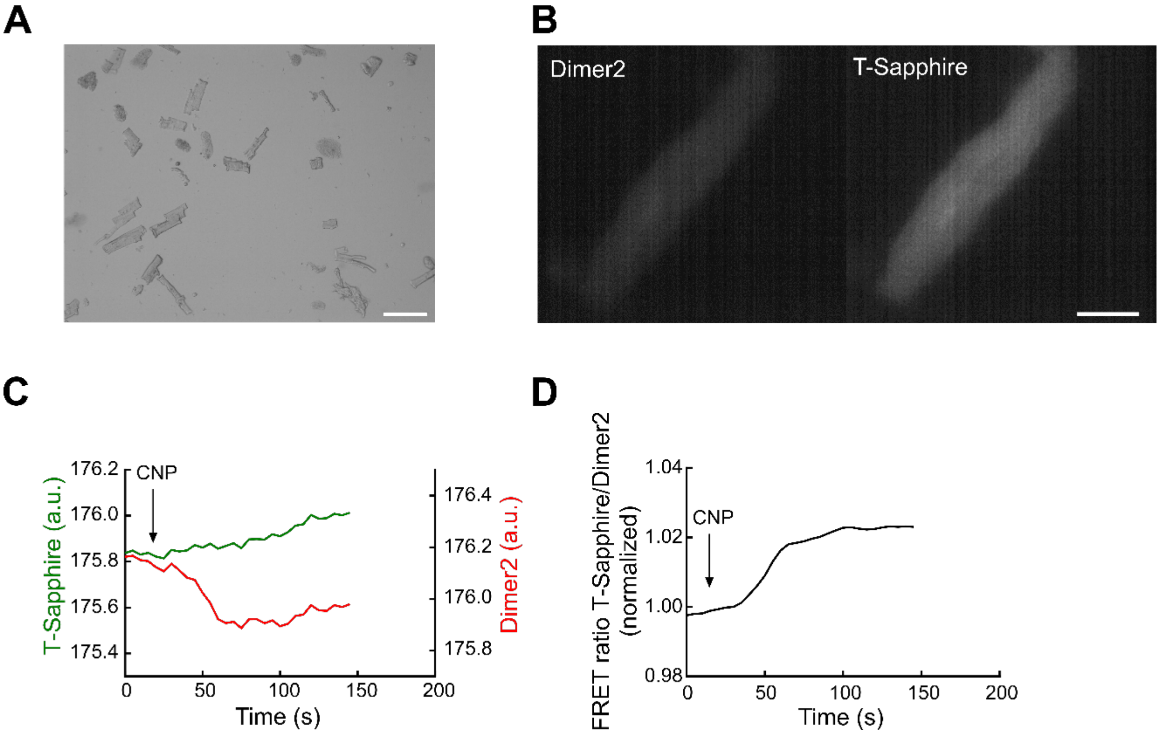

Supplementary Fig S7. Establishment of cGMP-FRET measurements in human ventricular myocytes. A, **B** Freshly isolated human ventricular myocytes (**A**), transmission light image, scale bar 500 µm were transduced with adenovirus to express red-cGES-DE5 biosensor, and (**B**) used for cGMP-FRET recordings 48h after transduction. T-Sapphire and Dimer2 fluorescence after successful sensor expression are shown. Scale bar 25 µm. **C**,**D** Representative cGMP-FRET recording upon stimulation with natriuretic peptide CNP (100 nM). Single fluorophore channels (**C**) and normalized T-Sapphire/Dimer2 ratio (**D**) are shown.

Fig. S8

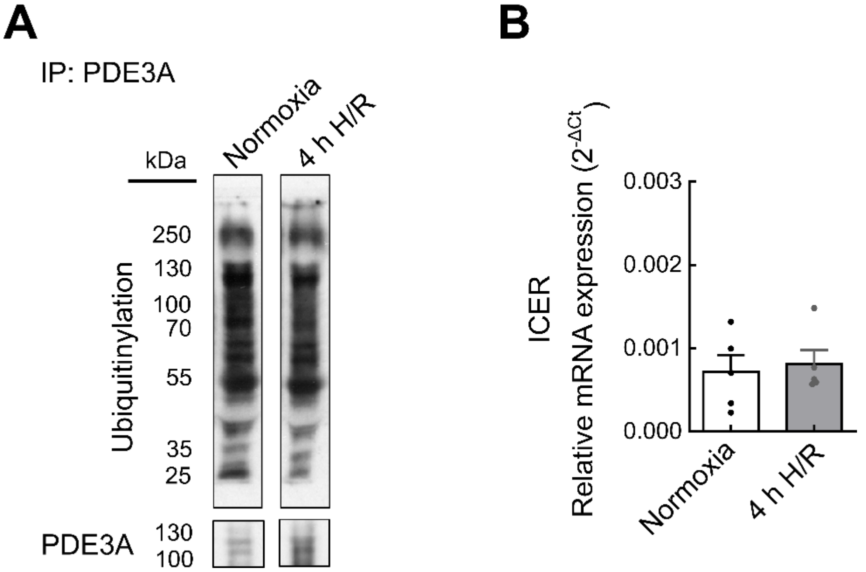

Supplementary Fig S8. Ubiquitinylation of PDE3A protein and inducible cAMP early repressor (ICER) expression after 4h H/R treatment. A Mono- and polyubiquitinylation of PDE3A protein in murine cardiomyocytes after 4h H/R or normoxia treatment. **B** Expression analysis of *Icer* gene in cardiomyocytes after 4h H/R or normoxia normalized to *Gapdh* (n=5 mice). Significance levels were tested with Mann-Whitney test. Neither changes in PDE3A ubiquitinylation nor in ICER expression can be detected at 4h H/R.

Fig. S9

A

GATTCCTGGCCTCCCAAGTGTGATTGGTGATCACGGCTCAGCAAGCGACTCCGGTGTGTAa
gcttaagctgggccctggtaat*ATAACTTCGTATAGCATACATTATACGAAGTTAT* ggctgctgcggaaacaacctacttga
gggcGGAACGCCAATTGTGTGTTGTATTTGTGGTTTGCATGTTGTATTTTCAAGTCAGGTAAT
CCCAGCTTCACAGTTTTAAAGAATGCCTTTCACTTTCACTTCCTCTCCTCAG ATTCTGACAG
TGGATTTACACACGGACACATGGGATATGTGTTTTCAAAAATGTATCACGTGCCAGATGA
CAAATATGGATGCCTGTCTGGGAATATTCCAGCCCTAGAGTTGAT GGCTCTGTACGTTGCT
GCAGCCATGCATGACTACGATCACCCAGGGAGGACAAATGCCTTCCTGGTTGCCACTAGT
GCCCCTCAGGTAAAGGCTTAGATTTTCGTGAGGAGATAATAATTTAGGTTCTACATTTTTT
GGAATAACATTAATTTCACAAATATGATGTATAGACACCAAACTGTAggatccaggttaggatcgcata
gg*ATAACTTCGTATAGCATACATTATACGAAGTTAT* ggctgctgctccttaatgcgcgtagtcgTTAATTTCCAAA
GATGTCATGGTTTGTGCCAAGACAATCATGATAGATATTTAACCTCAT

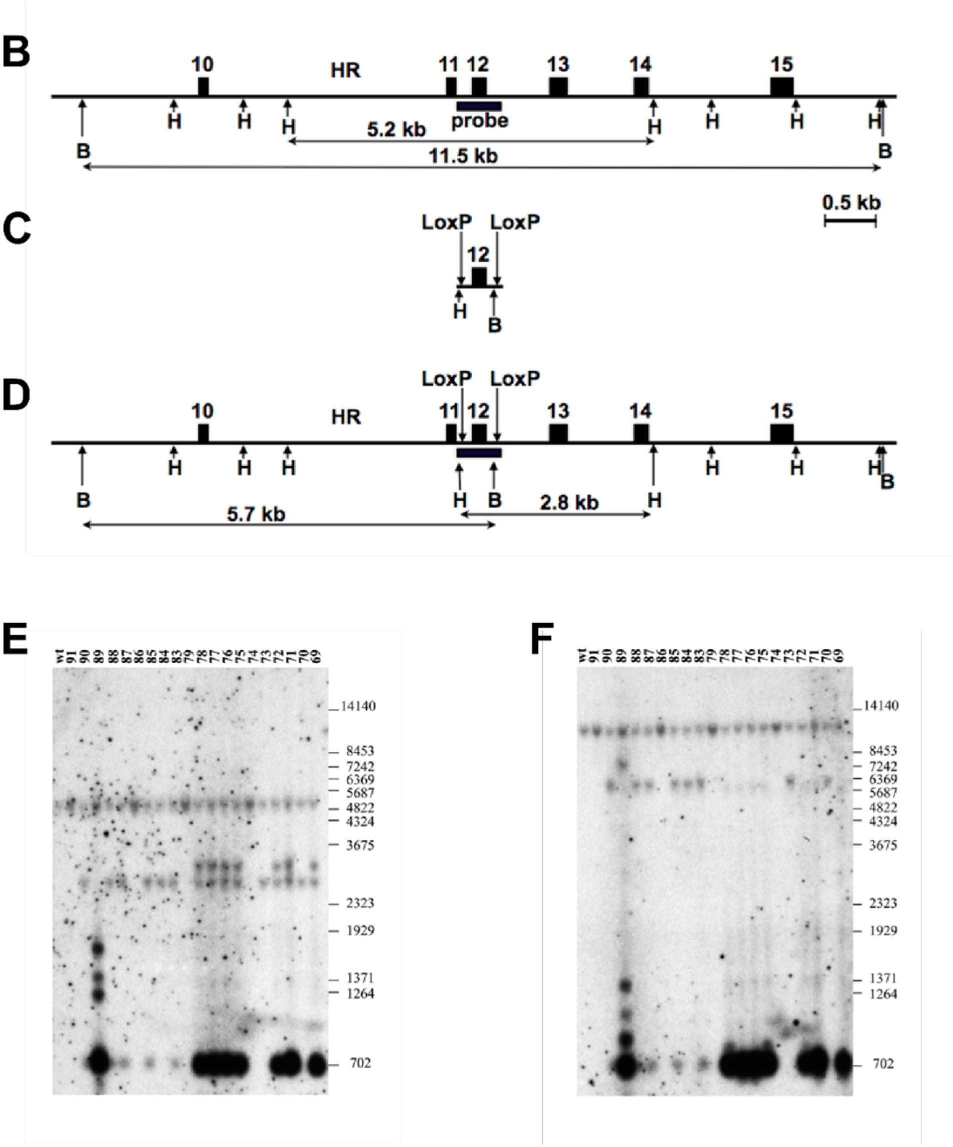

Supplementary Fig S9. Generation of the Pde3a conditional KO mouse model. A Sequence of mouse Pde3a donor DNA template. Capital letters represent the sequence of exon 12 – intronic region of mouse *Pde3a* gene. The nucleotides of exon 12 are underlined. Small letters represent artificially introduced sequences. The *Lox*P sites are indicated as capital italic letters. **B-D** Targeting

strategy of the exon 12 of mouse *Pde3a* gene. The intron regions are shown as line, exons are shown as filled boxes numbered above. The vertical arrows denote the two *Lox*P sites, and arrows below indicate restriction endonuclease sites *Hind*III (H) and *Bam*HI (B). The horizontal black bars designated as “probe” correspond to area hybridized with donor DNA specific probe used for Southern probe analysis. The expected sizes of restriction DNA fragments are labelled below in kb. (**B**) Wild type locus. (**C**) Schematic representation of *Pde3a* donor DNA template. (**D**) Genomic locus after the homologous recombination. **E, F** Southern blot analysis of F1 offspring. Southern blot analysis of genomic DNA isolated from mouse tail biopsy (69-79, 83-91) and hybridized with the Pde3a template DNA probe. (**E**) *Hind*III enzymatic digestion revealed the wild type allele (5.2 kb) and targeted allele at (2.8 kb) (B-D). The 0.7 kb band indicate the DNA fragment digested from head-to-tail (HtT) integrated donor DNA template. DNA samples obtained from mice numbers 70, 73 and 84 contain correctly targeted *Pde3a* allele at 5’ region. Positions of the size marker (in bp) are shown on the right. (**F**) *Bam*HI enzymatic digestion, to prove F1 mice with correctly targeted allele. The wild type allele corresponds to band 11.5 kb and targeted allele located to 5.7 kb (B-D). The 0.7 kb band corresponds to (HtT) insertion of DNA templates. DNA samples 70, 73 and 84 revealed correctly targeted Pde3a allele at 3’ region.

SUPPLEMENTARY REFERENCES

[1] B.V. Skryabin, D.M. Kummerfeld, L. Gubar, et al., Pervasive head-to-tail insertions of DNA templates mask desired CRISPR-Cas9-mediated genome editing events, Sci. Adv. 6 (7) (2020), https://doi.org/10.1126/sciadv.aax2941.

[2] S. Schobesberger, P.T. Wright, C. Poulet, J.L., et al., beta3-Adrenoceptor redistribution impairs NO/cGMP/PDE2 signalling in failing cardiomyocytes, Elife 9 (2020), https://doi.org/10.7554/eLife.52221.

[3] S. Börner, F. Schwede, A. Schlipp, et al., FRET measurements of intracellular cAMP concentrations and cAMP analog permeability in intact cells, Nat. Protoc. 6 (4) (2011) 427-438, https://doi.org/10.1038/nprot.2010.198

[4] K. Götz, J. Sprenger, R.K. Perera, J.H. Steinbrecher, S.E. Lehnart, M. Kuhn, et al., Transgenic Mice for Real Time Visualization of cGMP in Intact Adult Cardiomyocytes, Circ. Res. 114 (8) (2014), 1235–1245. https://doi.org/10.1161/CIRCRESAHA.114.302437.

[5] D. Calebiro, V.O. Nikolaev, M.C. Gagliani, T. de Filippis, et al., Persistent cAMP-signals triggered by internalized G-protein-coupled receptors, PLoS Biol. 7 (8) (2009) e1000172, https://doi.org/10.1371/journal.pbio.1000172.

[6] C. Jungen, K. Scherschel, C. Eickholt, P. Kuklik, et al., Disruption of cardiac cholinergic neurons enhances susceptibility to ventricular arrhythmias, Nat. Commun. 8 (2017) 14155. https://doi.org/10.1038/ncomms14155.

[7] C. Belge, J. Hammond, E. Dubois-Deruy, B. Manoury, et al., Enhanced expression of beta3-adrenoceptors in cardiac myocytes attenuates neurohormone-induced hypertrophic remodeling through nitric oxide synthase, Circulation 129 (4) (2014) 451-462. https://doi.org/10.1161/CIRCULATIONAHA.113.004940.
