# Supplementary material for: Rise of cGMP by partial phosphodiesterase-3A degradation enhances cardioprotection during hypoxia
Source: Redox Biol. 2021 Nov 6;48:102179. doi: 10.1016/j.redox.2021.102179 (PMC8590074; doi:10.1016/j.redox.2021.102179)

Full unedited immunoblots for Figure 1A

A

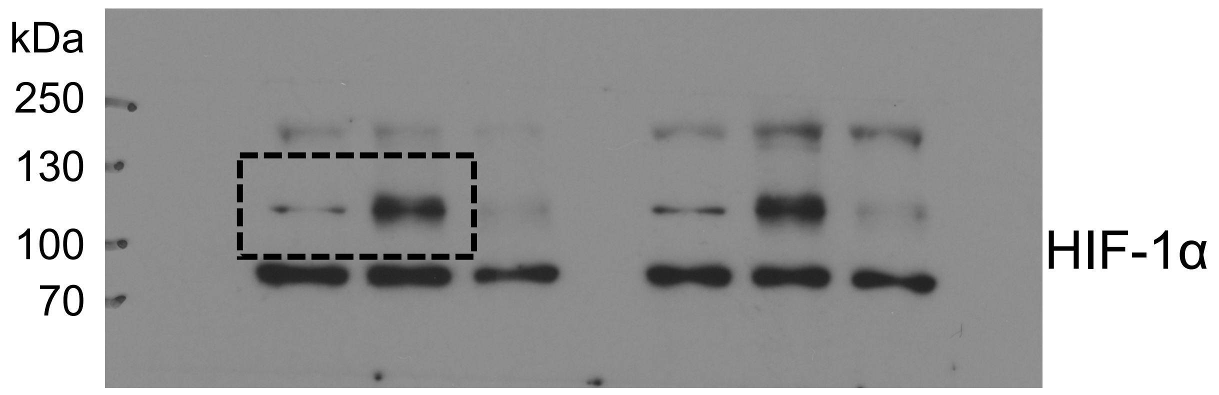

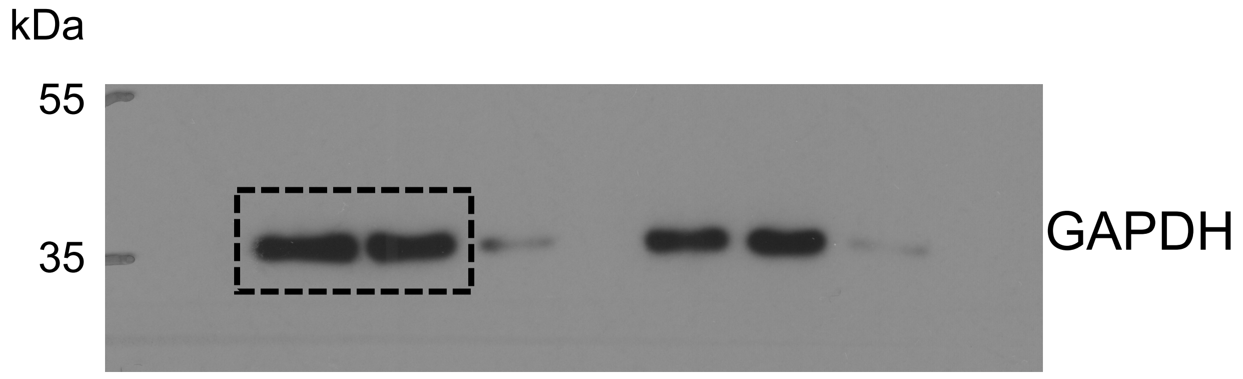

Full unedited immunoblots for Figure 2

A

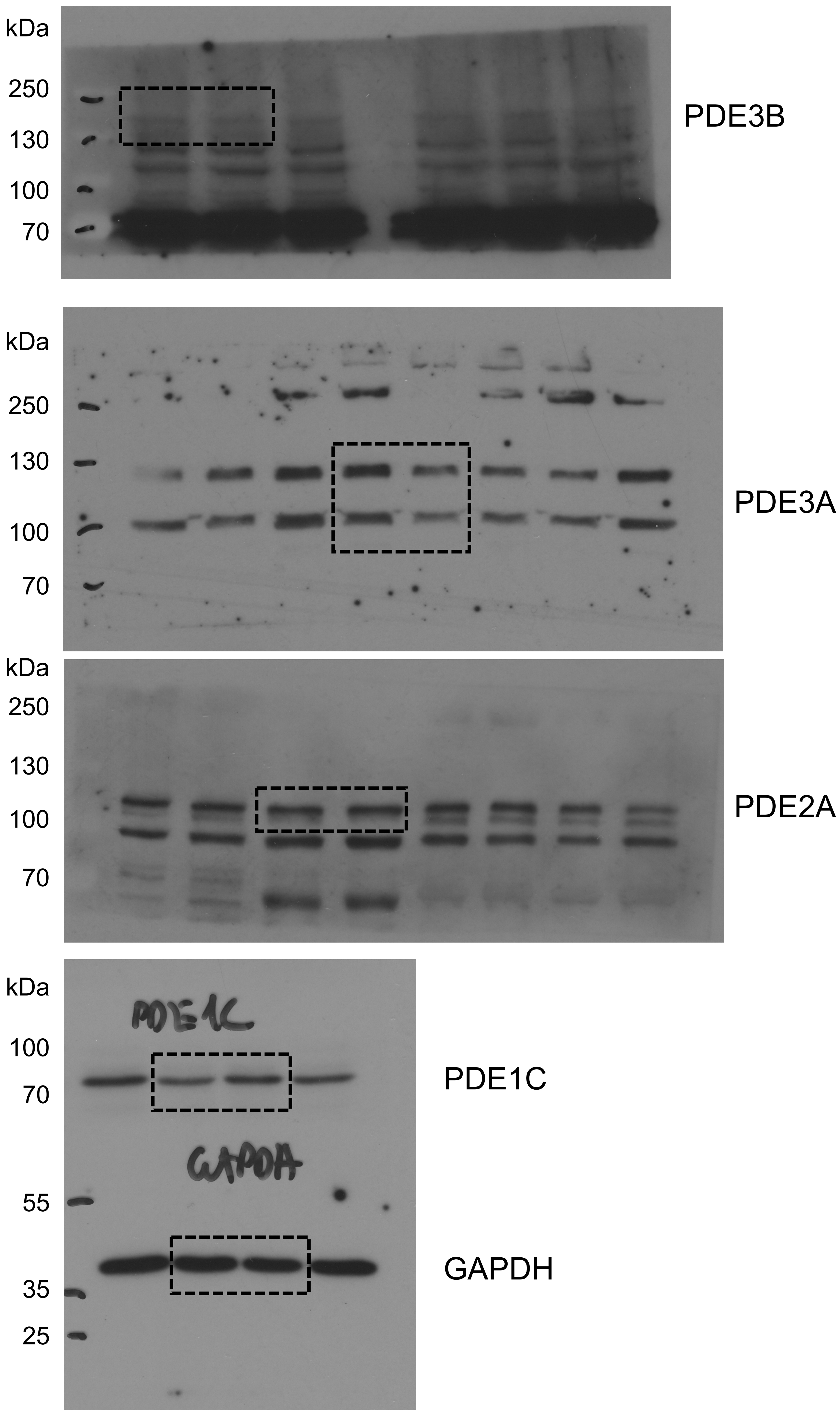

Full unedited immunoblots for Figure 2

G

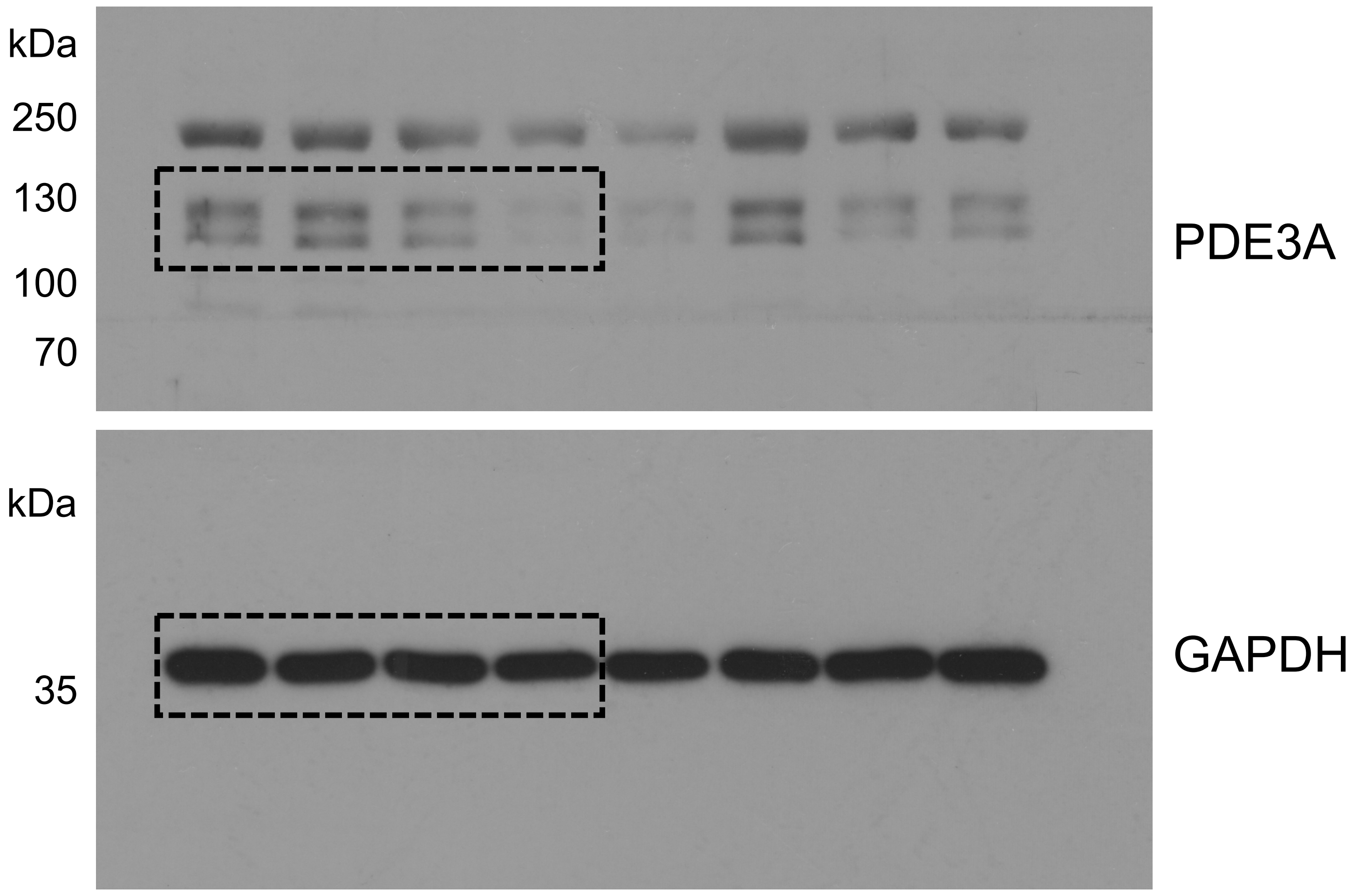

Full unedited immunoblots for Figure 3

G

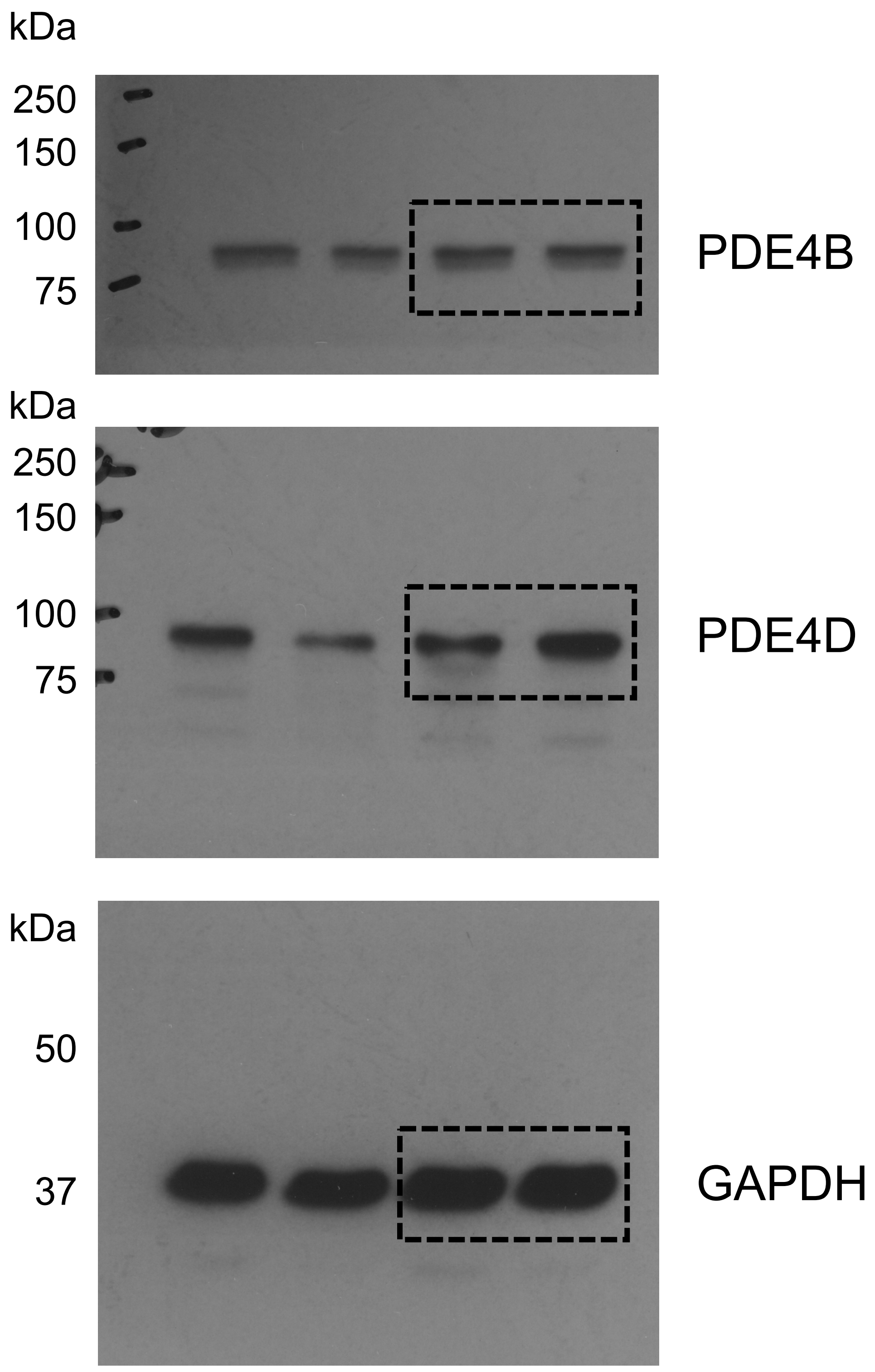

Full unedited immunoblots for Figure 4

B

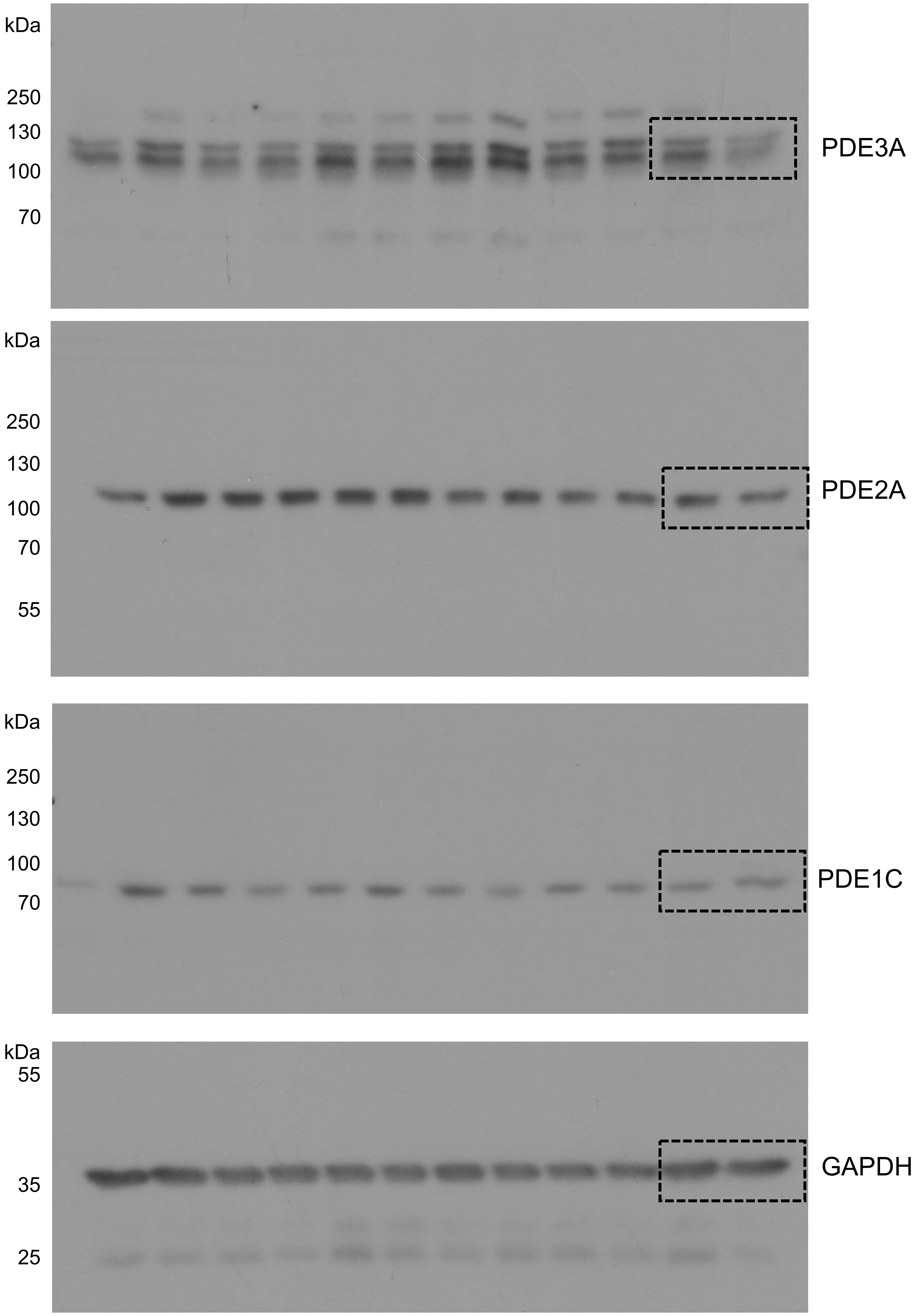

Full unedited immunoblots for Figure 5

E

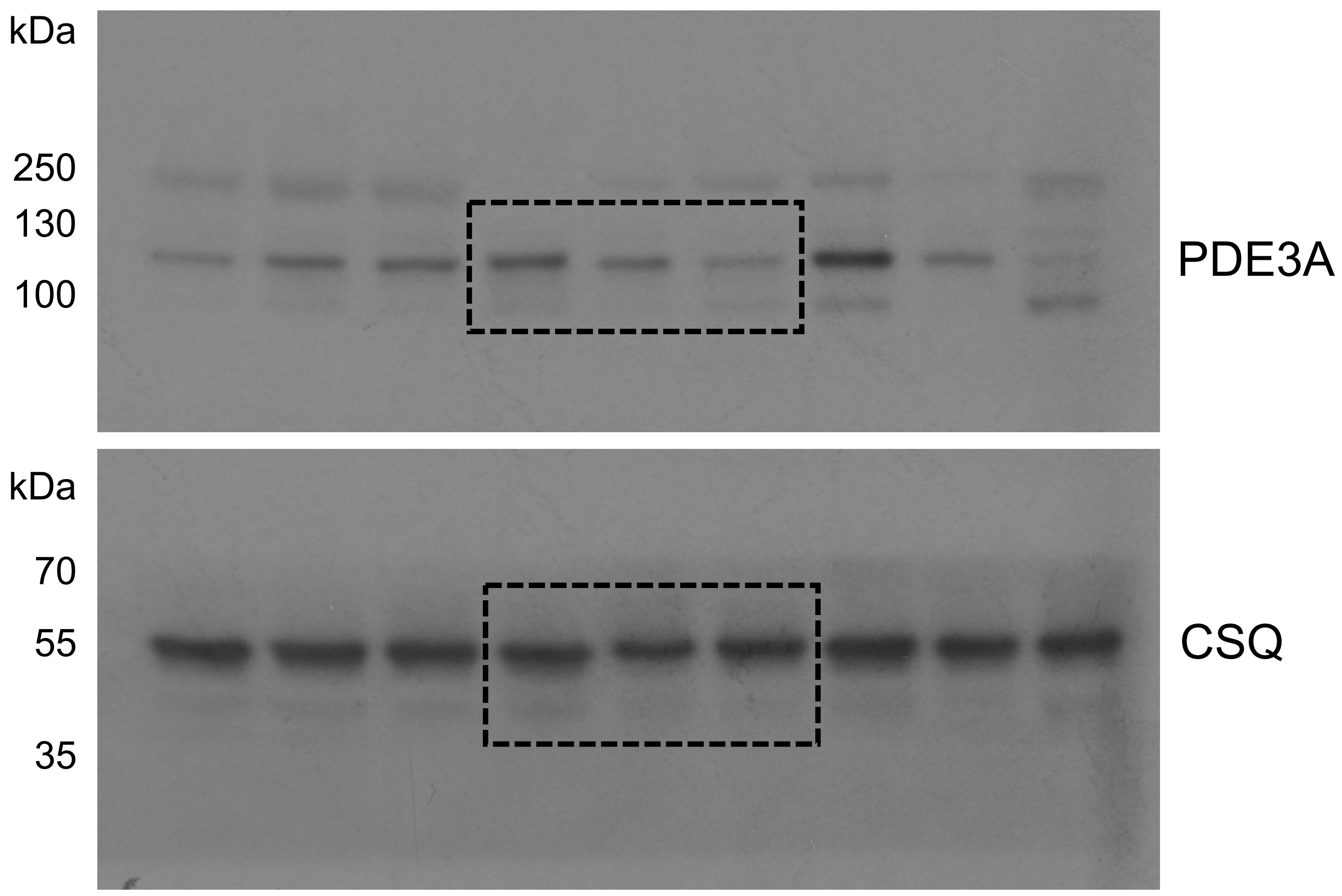

Full unedited immunoblots for Figure 6

A

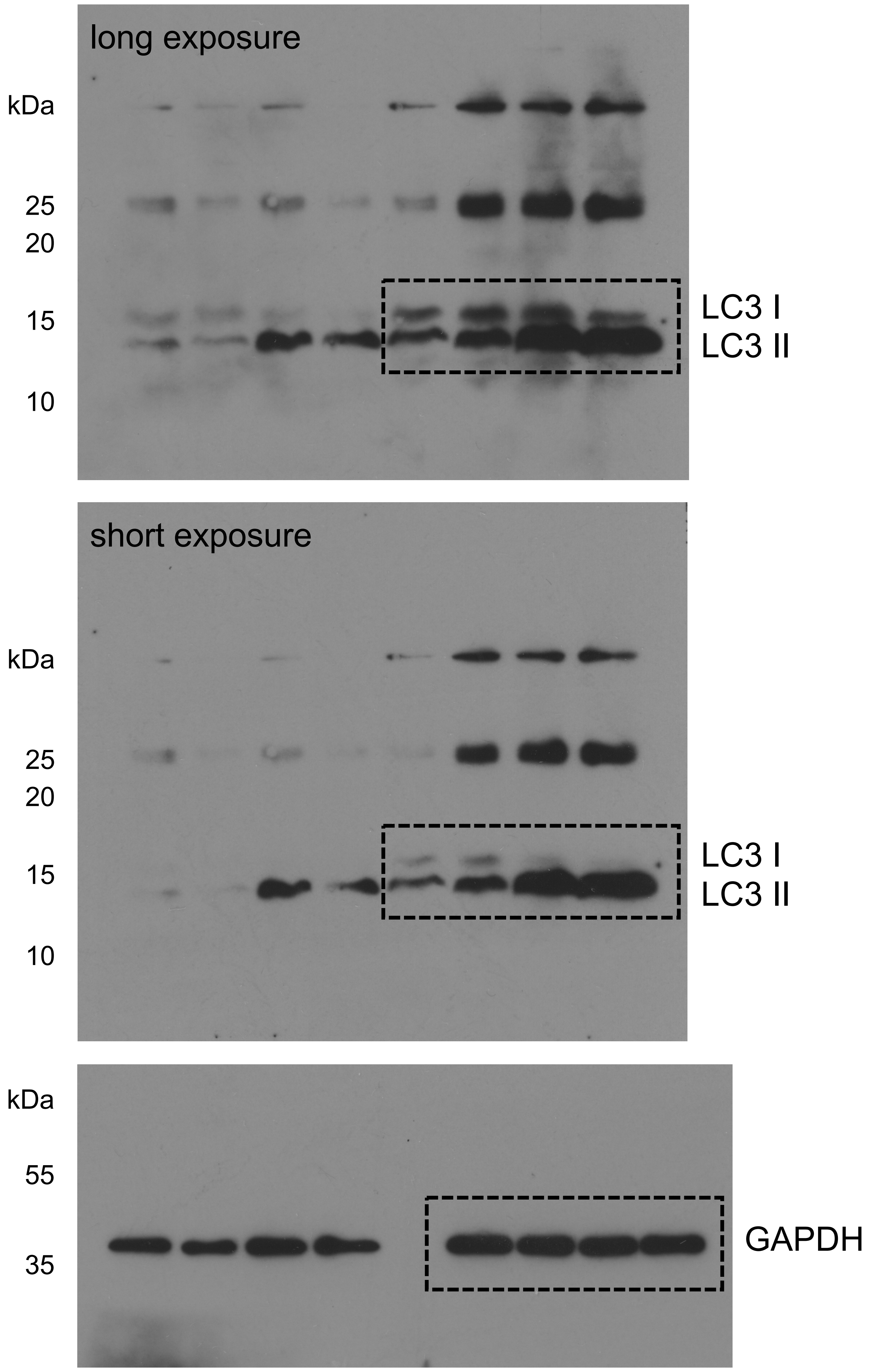

Full unedited immunoblots for Figure 6

B

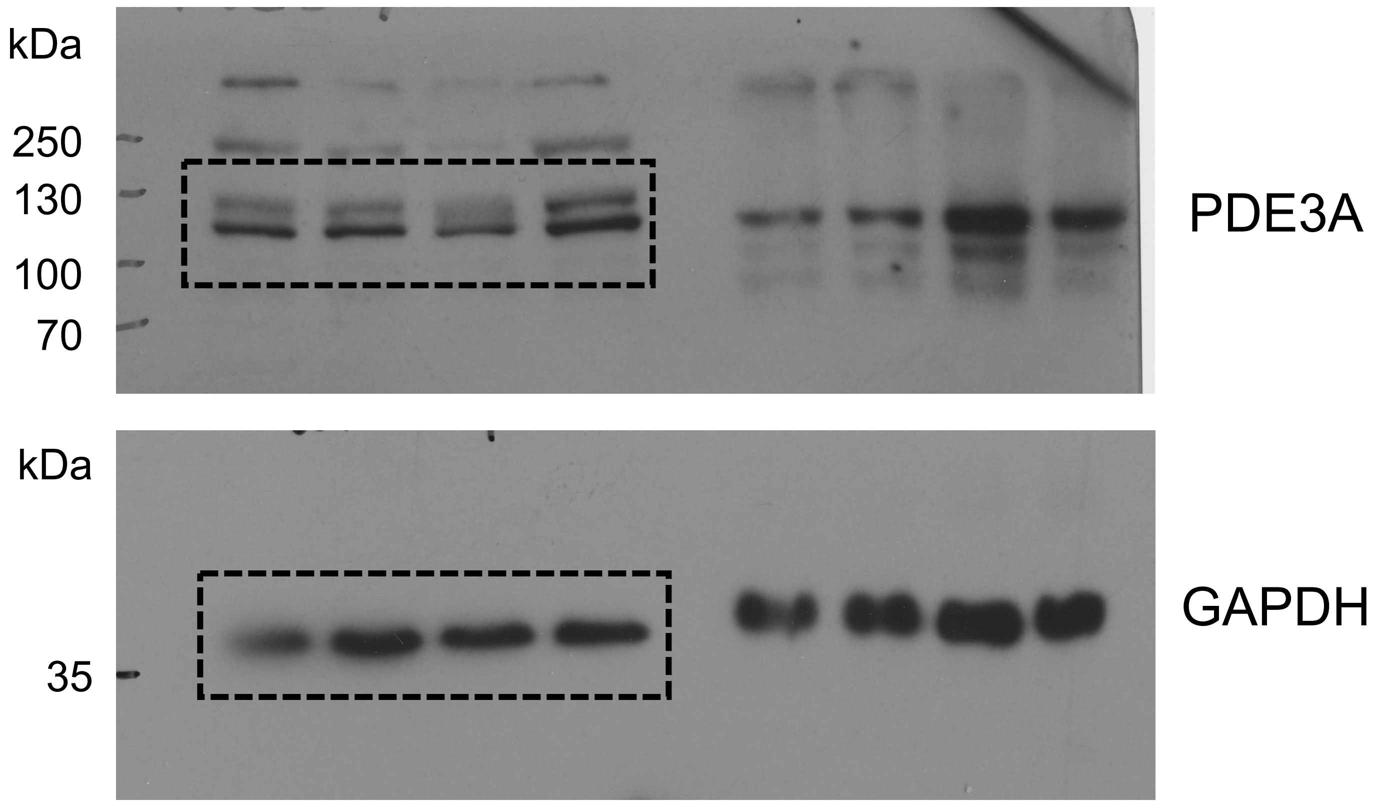

Full unedited immunoblots for Figure 7

A

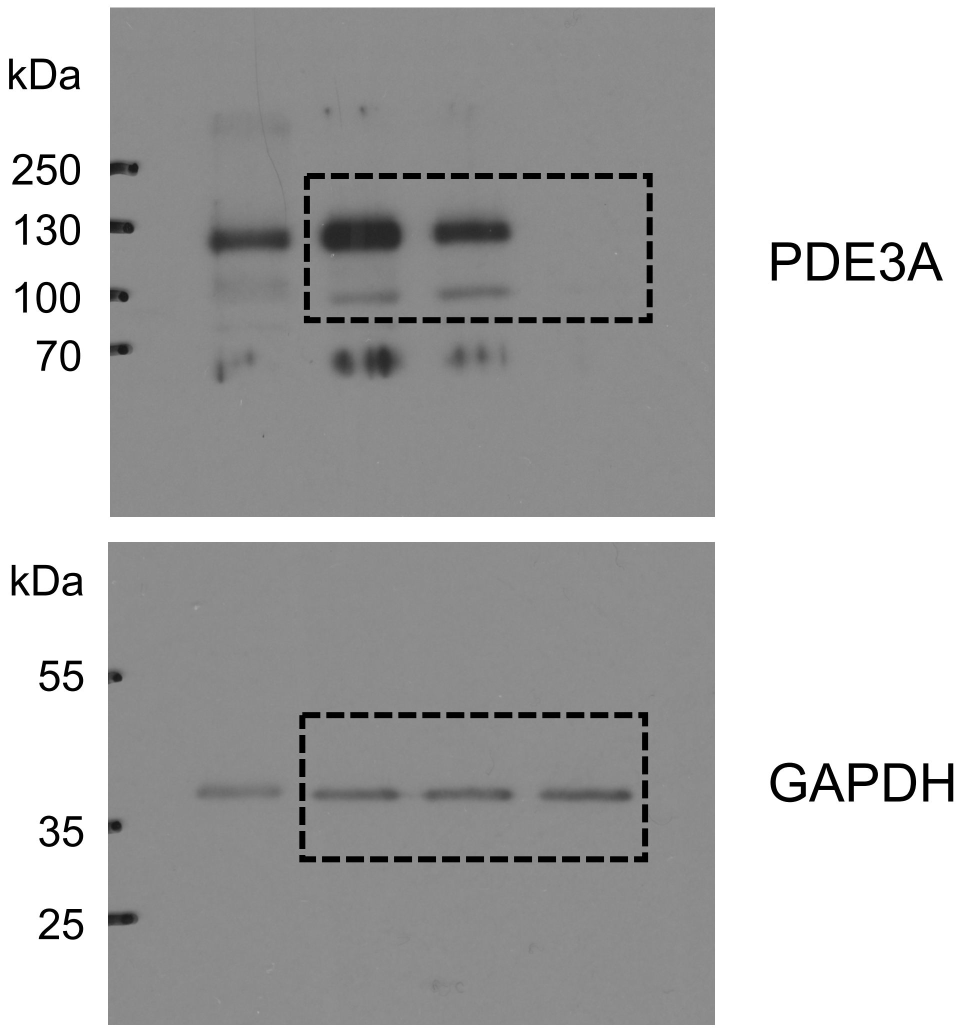

Full unedited immunoblots for Supplemental Figure 2

A

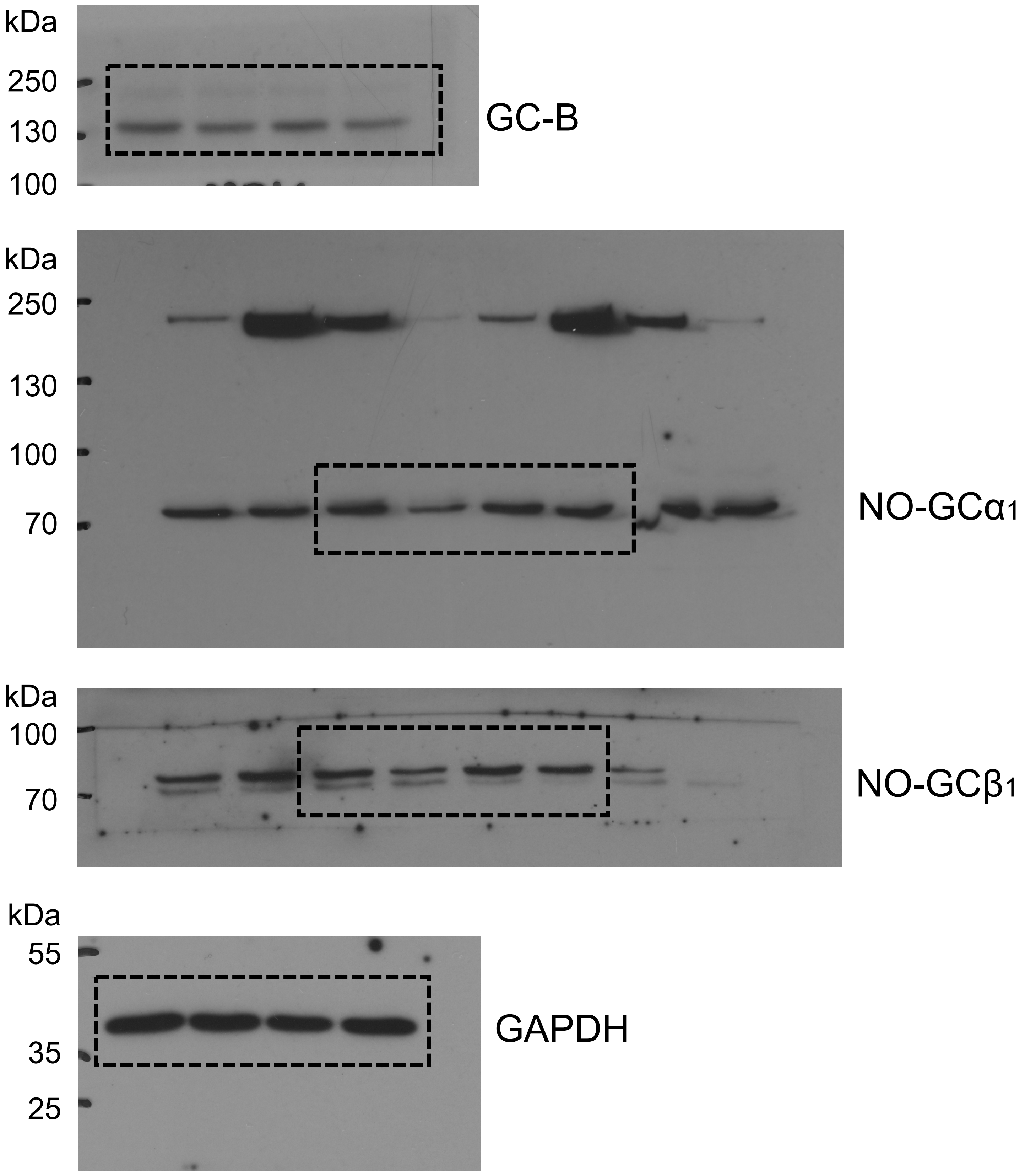

Full unedited immunoblots for Supplemental Figure 3

D

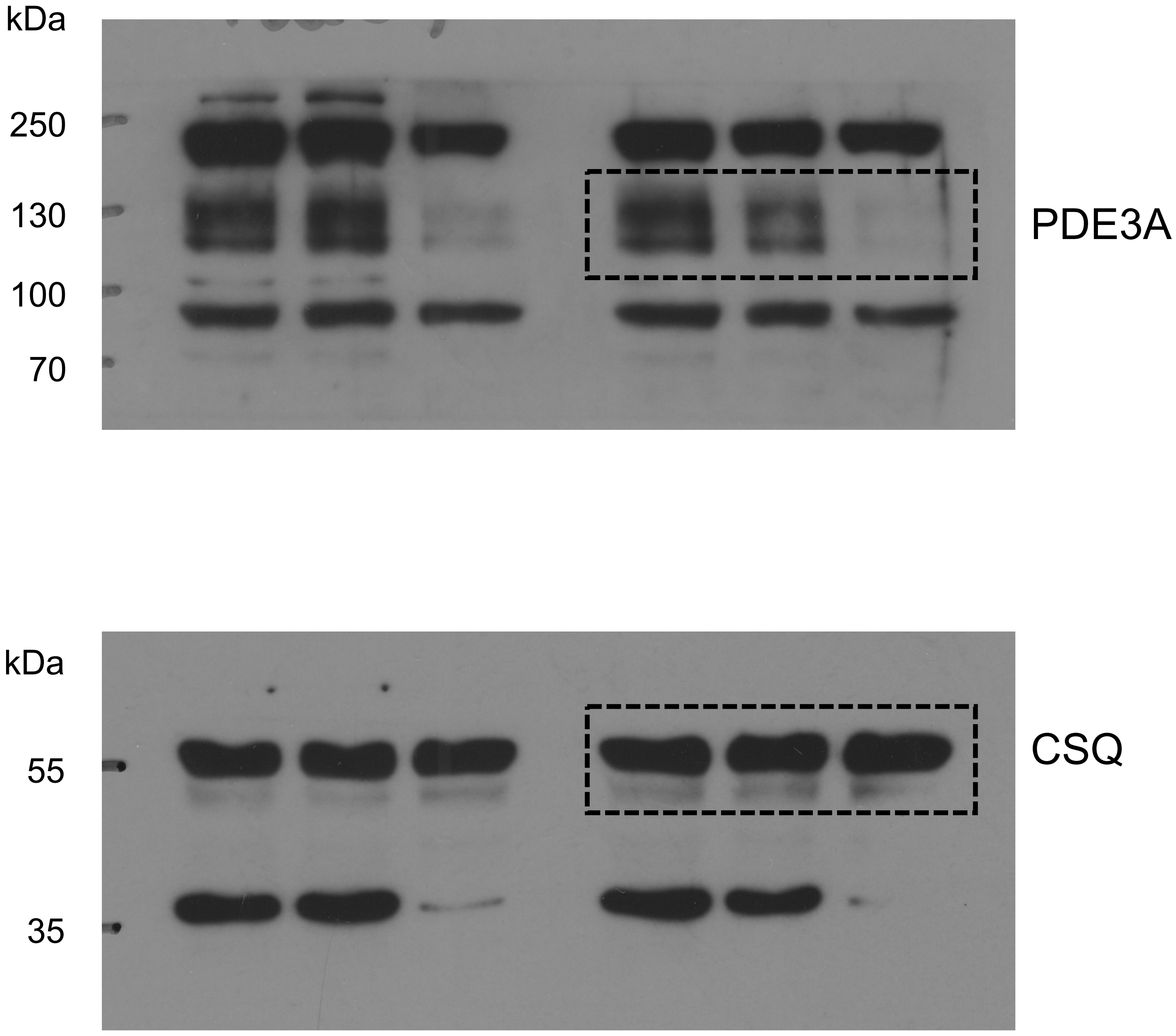

Full unedited immunoblots for Supplemental Figure 8

A

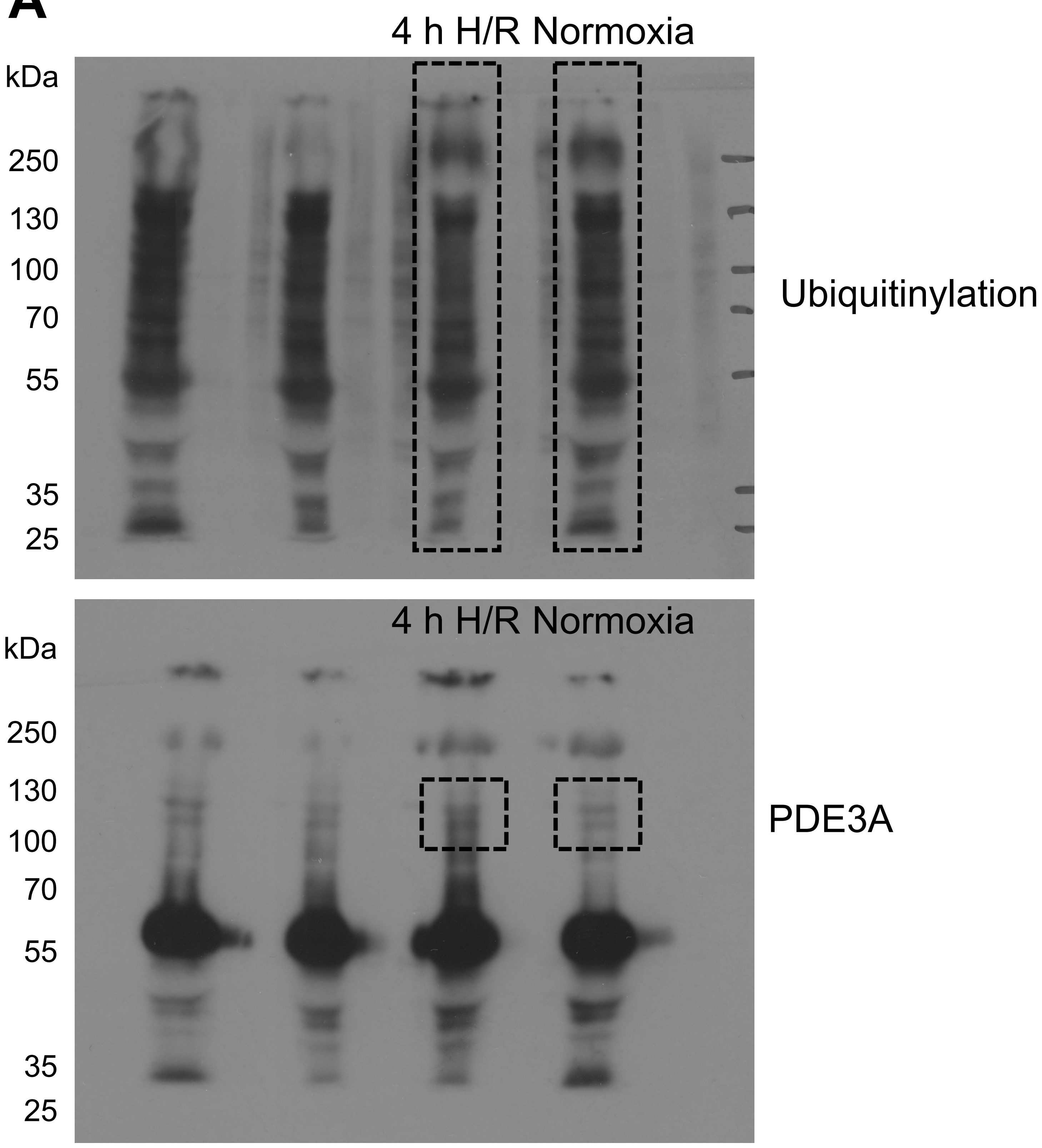

Supplement: Multimedia component 2 [file mmc2.pdf]
